# Effect of electronic adherence monitoring on adherence and outcomes in chronic conditions: A systematic review and meta-analysis

**DOI:** 10.1371/journal.pone.0265715

**Published:** 2022-03-21

**Authors:** Amy Hai Yan Chan, Holly Foot, Christina Joanne Pearce, Rob Horne, Juliet Michelle Foster, Jeff Harrison

**Affiliations:** 1 School of Pharmacy, Faculty of Medical and Health Sciences, The University of Auckland, Auckland, New Zealand; 2 Centre of Behavioural Medicine, School of Pharmacy, University College London, London, United Kingdom; 3 Woolcock Institute of Medical Research, University of Sydney, Sydney, Australia; Universidad de Antioquia, COLOMBIA

## Abstract

**Introduction:**

Electronic adherence monitoring (EAM) is increasingly used to improve adherence. However, there is limited evidence on the effect of EAM in across chronic conditions and on patient acceptability. We aimed to assess the effect of EAM on adherence and clinical outcomes, across all ages and all chronic conditions, and examine acceptability in this systematic review and meta-analysis.

**Methods:**

A systematic search of Ovid MEDLINE, EMBASE, Social Work Abstracts, PsycINFO, International Pharmaceutical Abstracts and CINAHL databases was performed from database inception to December 31, 2020. Randomised controlled trials (RCTs) that evaluated the effect of EAM on medication adherence as part of an adherence intervention in chronic conditions were included. Study characteristics, differences in adherence and clinical outcomes between intervention and control were extracted from each study. Estimates were pooled using random-effects meta-analysis, and presented as mean differences, standardised mean differences (SMD) or risk ratios depending on the data. Differences by study-level characteristics were estimated using subgroup meta-analysis to identify intervention characteristics associated with improved adherence. Effects on adherence and clinical outcomes which could not be meta-analysed, and patient acceptability, were synthesised narratively. The Preferred Reporting Items for Systematic Reviews and Meta-analyses (PRISMA) guideline was followed, and Risk of bias (RoB) assessed using the Cochrane Collaboration’s RoB tool for RCTs. The review is registered with PROSPERO CRD42017084231.

**Findings:**

Our search identified 365 studies, of which 47 studies involving 6194 patients were included. Data from 27 studies (n = 2584) were extracted for the adherence outcome. The intervention group (n = 1267) had significantly better adherence compared to control (n = 1317), (SMD = 0.93, CI:0.69 to 1.17, p<0.0001) with high heterogeneity across studies (I^2^ = 86%). There was a significant difference in effect according to intervention complexity (p = 0.01); EAM only improved adherence when used with a reminder and/or health provider support. Clinical outcomes were measured in 38/47 (81%) of studies; of these data from 14 studies were included in a meta-analysis of clinical outcomes for HIV, hypertension and asthma. In total, 13/47 (28%) studies assessed acceptability; patient perceptions were mixed.

**Interpretation:**

Patients receiving an EAM intervention had significantly better adherence than those who did not, but improved adherence did not consistently translate into clinical benefits. Acceptability data were mixed. Further research measuring effects on clinical outcomes and patient acceptability are needed.

## Introduction

Poor medication adherence costs the US health system between $100 and $300 billion of avoidable health care costs annually, and is associated with increased morbidity and mortality [1, 2]. Despite the large body of literature on adherence, medication adherence remains suboptimal [1]. Interventions to improve adherence have had only modest impacts on adherence, and have uncertain long-term sustainability due to the short trial durations and need for intensive resources [1, 3]. Digital solutions can address some of these concerns by potentially improving intervention sustainability through automation and reduce resources for implementation [4]. Exploring new ways of delivering healthcare is essential with the COVID-19 pandemic and increased pressures on health systems [5–7].

Electronic adherence monitoring (EAM) [4] use electronic devices that record medication-taking, usually the time and date of each dose. These medication monitors are increasingly used as part of strategies to improve adherence. EAM is seen as the gold standard of adherence measurement due to their objectivity and data recording accuracy [4, 8], and can be used to improve adherence through direct patient reminders for medication-taking [9], and/or by facilitating adherence feedback to the patient [10, 11]. Previous reviews have looked at the effect of certain features of EAM and associated electronic devices such as reminders [7, 12–15], medication packaging [13], or adherence feedback [10], on adherence. The reviews generally report a positive effect on adherence [7, 12–15] however no reviews have examined EAM specifically, or across all chronic conditions rather than specific conditions [14, 15]. Christensen et al. for example conducted a systematic review of studies on EAM for oral antihypertensive medicines, and found that most reported average adherence rates above 80%, though adherence did vary from 0 to 101% [16]. The authors did not perform a meta-analysis. In a systematic review and meta-analysis by Yaegashi et al., adherence as measured by EAM was reported to be 71% for antipsychotics in schizophrenia [17]. Lee et al. conducted a meta-analysis of RCTs of EAM in children with asthma and reported that the EAM group was 1.50 times more likely to adhere to inhalers compared with the control group [18]. However, these reviews have not included clinical outcome data [13], or where there is outcome data, the review has not been systematic [9] or did not include a meta-analysis [10], or focused on specific populations or medication [17, 18].

Given the costs of poor adherence, and the increasing investment into EAM to improve adherence, there is a need for a high quality systematic review and meta-analysis. The findings will inform public health decision-making and future strategies to improve adherence and outcomes across chronic conditions. This systematic review examines the effect of EAM across all chronic conditions on adherence and clinical outcomes.

## Materials and methods

This systematic review was conducted based on *Guidelines of the Cochrane Collaboration* as described in the *Cochrane Handbook of Systematic Reviews of Interventions*, version 6.0 (updated July 2019) [19] and the Preferred Reporting Items for Systematic Reviews and Meta-Analyses (PRISMA). The review is registered with PROSPERO CRD42017084231.

### Search strategy and selection criteria

We did a systematic search of the literature using Ovid Medline, EMBASE, Social Work Abstracts, PsycINFO, International Pharmaceutical Abstracts, EBM Reviews–Cochrane Central Register of Controlled Trials and CINAHL from database inception to June 1, 2020. Indexing terms based on electronic monitoring, adherence, and intervention were used to develop the search strategy; full details are in S1 Appendix. No language or participant type limit was used. This search strategy was supplemented by a manual search of the reference lists of the identified studies to find other relevant studies. All titles and abstracts were screened separately by 2 authors independently (AC first, with a second screen by JH/CP/HF). Full texts were obtained for eligible studies or abstracts that did not have sufficient information for review. Studies that did not meet the inclusion criteria or had reasons for exclusion were not reviewed further, and reasons for exclusion documented.

Inclusion criteria were: a) the intervention evaluated EAM as an intervention to improve medication adherence; b) participants were individuals with chronic conditions, defined as a long-term, persistent health condition lasting 3 months or more [20]; c) one of the outcome measures was medication adherence, though this did not need to be measured by electronic monitoring, and clinical outcomes did not need to be assessed; and d) the study was a RCT or a controlled clinical trial (to ensure the highest quality of evidence was included). Studies were still eligible for inclusion even if only one group had adherence monitored electronically and the other did not. Cluster trials were eligible for inclusion. EAM was defined as any mechanism or device that measures and records adherence electronically, regardless of whether or not the EAM devices had a reminder function. All studies meeting the inclusion criteria were included regardless of how adherence was measured (via EAM or not), the adherence measurement (objective or self-report), definition of adherence (taking, timing adherence, or difference between two time-points) or analysis method (e.g. mean ± SD, odds ratios). Adherence had to be measured for all participants at the individual level and not at a group level e.g. adherence of individual patients not adherence of patients within one pharmacy site. Studies that used a downstream measure to approximate adherence e.g. adherence knowledge as a proxy for adherence, or used electronic monitoring for adherence measurement only, rather than to improve adherence, were excluded. Studies using a within-subjects design or historical controls were excluded due to the risk of bias arising from factors other than the intervention itself. Studies using contemporary controls were included.

### Data analysis

AC and HF extracted the following data for each study: general study information (author, year of publication); study design; study population (age, sex, health condition); study duration; type of EAM used; description of the intervention and control conditions including details on intervention complexity (i.e. how many components were included in the intervention in addition to EAM e.g. whether the intervention used EAM alone, EAM + EAM reminder, or EAM + health professional input, or all of the aforementioned components); method of adherence feedback; timing of the adherence feedback to the individual (immediate or delayed); presence of participant blinding to adherence monitoring function of EAM; how adherence was measured; outcome measures–effect on adherence, clinical outcomes and other findings; and any data on patient perceptions of EAM.

Studies were classified based the chronic condition of the participants in the study, and on how EAM were used in the adherence intervention–either *direct-to-patient* to improve adherence (e.g. via a reminder or visual feedback), or through an *indirect provider-to-patient* interaction (e.g. adherence feedback by the health provider), or *both*. The effect on clinical outcomes, where reported, was classified as “significantly improved”, “trend towards improvement but not significant”, “no effect”, or “worsened”. Patient perceptions of the EAM intervention were categorised as perceptions of EAM or of the adherence intervention.

GRADE was used to rate the quality of evidence according to risk of bias, consistency, directness, precision and reporting bias [21]. The risk of bias (RoB) in each included study was assessed independently, using the Cochrane Collaboration’s RoB tool for RCTs [22], by AC and HF/CP. A funnel plot was used to evaluate the effect of publication bias.

Data were pooled from studies which reported medication adherence of participants in the intervention and control groups. The primary outcome measure was the difference in medication adherence between intervention and control groups, expressed as the mean difference (MD) with 95% confidence intervals (CIs), and derived using random-effects models to account for both within-study and between-study variance (tau-squared [τ^2^]).SMD was used to account for different measures of adherence reporting. The SMD expresses the intervention effect in standard units rather than the original units of measurement and shows the difference in mean effects between the intervention and control groups divided by the pooled standard deviation of participants’ outcomes [19]. A positive SMD (i.e. greater than 0) indicates better adherence in the intervention group compared to control. To accommodate for differences between studies in adherence measures, adherence definitions, and analysis methods, the generic inverse variance outcome type was used. All estimates are presented as SMDs. As medication adherence differs significantly among different health conditions, the adherence outcome was analysed by chronic disease. There were two secondary outcomes: the difference in medication adherence between intervention and control groups in studies that a) measured taking adherence (i.e. studies that measured the percentage of prescribed doses taken, regardless of timing [23]) and b) studies that used an objective measure of adherence.

We contacted authors for studies that did not have data or could not be converted into the required format for meta-analysis (i.e. SMD and standard deviation). The primary outcome measure was chosen for studies that had multiple medications or dosing regimens or adherence measures (e.g. timing and taking adherence); or reported multiple intervention or control groups. If outcomes were reported at multiple time points, we extracted these and included the latest reported time point. We excluded post-intervention follow-up data. If multiple measures of adherence were used, we included the most objective measure in the review. Reporting in the study of one or more of the outcomes listed here was not an inclusion criterion for this review. Intention-to-treat (ITT) or ’full analysis set’ analyses were used where these were reported. For studies that did not report data in a form that allowed meta-analysis, this data were reported narratively (e.g. as medians and interquartile ranges for each group).

Studies that included all as primary or secondary outcomes, decisions were made in the following order: the most recent (or end) time-point in the intervention period; the group with the largest number of participants; the dosing regimen with the least daily doses; taking adherence; and the intervention group that most closely represented EAM alone and the control group that most closely represented usual care.

The Ԛ test [24] and the I^2^ index were used to identify and quantify study heterogeneity respectively. Cochrane RevMan Software version 5.4 [25] was used for all statistical analyses, and p-values <0.05 denoted statistical significance.

#### Study-level characteristics: Subgroup analysis

We conducted pre-specified subgroup analyses to investigate the effect of the following study-level characteristics on adherence: 1) age; 2) healthcare setting; 3) intervention complexity (i.e. EAM alone, EAM + EAM reminder, EAM + health professional input or all aforementioned components); 4) method of adherence feedback; 5) timing of adherence feedback to the participant (immediate or delayed); 6) study duration; and 7) participant blinding to the EAM adherence monitoring function.

*Effect on clinical outcomes*. Based on the heterogeneity of the different disease measures, we conducted meta-analyses only when this was meaningful, that is, when treatments, participants, and the underlying clinical question were similar enough for pooling to make sense, for example, where studies used similar outcome measures. We therefore performed a meta-analysis by grouping together similar measure types according to the chronic disease. For studies that did not report data in a form that allowed

meta-analysis, this data were reported narratively (e.g. as medians and interquartile ranges for each group).

GRADE was used to rate the quality of evidence according to risk of bias, consistency, directness, precision and reporting bias [21]. The risk of bias (RoB) in each included study was assessed independently, using the Cochrane Collaboration’s RoB tool for RCTs [22], by AC and HF/CP. Funnel plots were used to evaluate the effect of publication bias.

Data were pooled from studies which reported the clinical outcome of interest in the intervention and control groups. Continuous data (data that can take any numerical value) was analysed as mean differences (MDs) using a random-effects model and 95% confidence intervals (CIs) if the measures used in the studies were reported on the same scale. If data were reported using different measures or scales, SMDs were used to account for the different methods of measurement (e.g. different asthma control questionnaires. If both change from baseline and endpoint scores were available for continuous data, change from baseline scores were used. For data reported as rates or proportions, this was analysed as risk ratios using a random-effects model and by inverse variance. If a study reported outcomes at multiple time points, we used the measure taken at the last follow-up. Intention-to-treat (ITT) or ’full analysis set’ analyses were used where these were reported.

*Patient acceptability of the EAM intervention*. Don patient acceptability were synthesised narratively.

## Results

**Fig 1:**
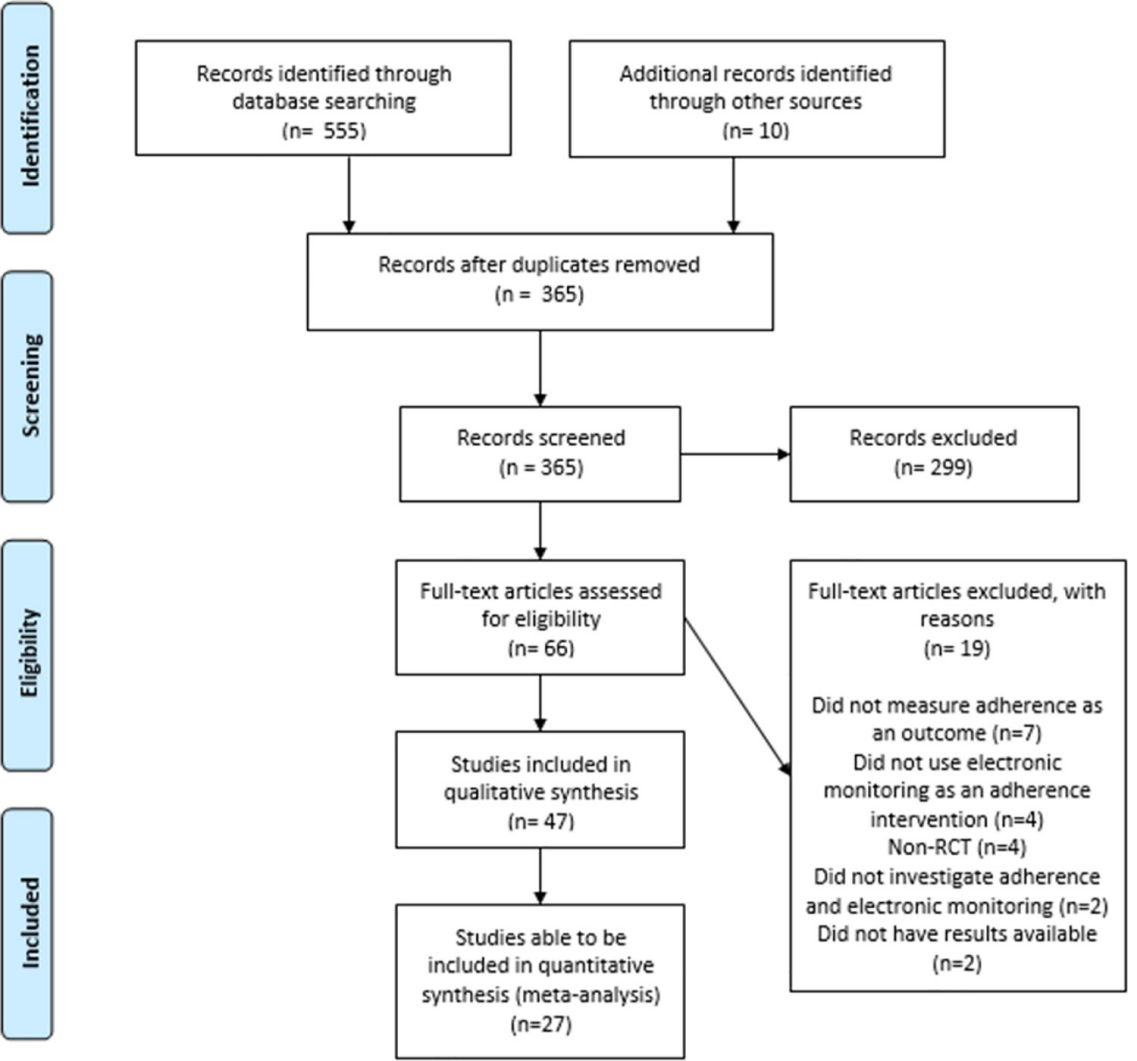
PRISMA flow diagram of eligible studies.

Our search identified 565 records, of which 365 were screened after duplicates were removed. 66 full-text articles were assessed for eligibility and 47 studies involving 6194 patients met the inclusion criteria for inclusion in this systematic review (Fig 1). Table 1 describes the main characteristics of the studies. Study population size ranged from 6 [26] to 784 [14] participants (mean = 128, median = 80). Most (n = 41, 87%) were in adults with only 6 studies in children. The most common conditions were in asthma (n = 10, 21%) [11, 27–35], or human immunodeficiency virus (HIV) (n = 9, 19%) [36–44], or hypertension (n = 6, 13%) [14, 45–49].

**Table 1:** Characteristics of included studies (n = 47).

Author	Year	Setting	Condition	Subjects	Length of study	Completion rate	Type of EAM	Intervention	Control
Andrade et al.	2005	The Johns Hopkins Moore HIV Clinic, Baltimore, MD	HIV	64 HIV-infected males and females ≥ 18 years attending HIV clinic	24 weeks	58/64	Electronic verbal prompting device—Disease Management Assistance System (DMAS). Device produces a timed, programmed voice message that prompts subjects to take medication. Records dosing times and dates when response button is pressed. Data can be uploaded and printed.	Same as control plus the DMAS device programmed with reminder messages and dosing times for each medication in the HAART regimen. Adherence results reviewed with participants.	Monthly 30-min adherence counselling session with education about barriers to adherence, hazards of non-adherence and their HAART regimen.
Artinian et al.	2003	Congestive Heart Failure (CHF) clinic of the Detroit Veterans Affairs Medical Centre	CHF	18 outpatients from the CHF clinic aged 50 to 87 years	3 months	18/18 (drop outs not discussed so assume 100% completion)	Medication compliance device—Med-eMonitor linked to a Web-based monitoring system via the patient’s telephone line. Stores up to 5 medicines and has an alarm to remind patients when to take their medication, which to take, and how many. Daily reminders given about healthy lifestyle, other medicines and questions about symptoms, blood pressure, weight. Records date and time stamp of pill compartment opening and patient responses to questions. Data uploaded daily to central server—accessible by clinicians.	Same as control plus a Med-eMonitor	Educational booklet on CHF self-care behaviours, cardiologist follow-up as per usual care, education from pharmacist about medication-taking and provision of written medication information
Brath et al.	2013	Diabetes outpatient clinic	Risk of cardiovascular conditions (≥2 of T2DM, Hypertension or hypercholesterolaemia)	150 patients with a defined risk for cardiovascular conditions	52 weeks	53/77	Medication adherence management system (mAMS): Electronic monitoring medication blister which connected with mobile phone app to send information about adherence to a web based telehealth system which was then used to remind patients automatically via text message	Electronic blister pack transmit user information via mobile phone and automatically sent feedback back to user via text message. Participants with < 70% adherence were also called once a week to try and increase adherence	Given a standard medication blister and returned to study group after control phase completed
Burgess et al.	2010	Paediatric asthma clinic within an outer metropolitan general hospital, Australia	Asthma	26 children aged between 6 and 14 years	4 months	26/26	Smartinhaler—validated EAM.	Same as control but had the measured adherence with preventive medication was fed back to the child, parent and physician then incorporated into the management plan for the next month.	Personalized asthma education and management plan, plus generic written information. Devices replaced monthly but adherence data not shared.
Chan et al.	2015	Regional hospital emergency department, Auckland, NZ	Asthma	220 children aged between 6 and 15 years	6 months	213/220	SmartTrack EAM with audiovisual reminder function for use with preventive medication. Records date, time and number of actuations used. Has 14 different reminders that ring twice daily, stopping after the correct dose is taken or after 15 min. Reminder only goes if the correct dose is not taken within 6 hours of the set reminder time.	SmartTrack EAM with audiovisual reminder function enabled (twice daily reminders)	SmartTrack EAM with audiovisual reminder function disabled
Charles et al.	2007	P3 research clinical trials facility, Wellington, NZ. Recruitment was from research volunteer databases, newspaper advertisements, informal contacts	Asthma	110 patients aged 12 to 65 years	2 week run-in period, 24 weeks after	90/110	Smartinhaler—EAM for use with pMDIs. It has an audiovisual reminder function, that emits an audible reminder (beep) at set times plus a visual cue to show patients whether they have taken their inhaler during a designated period or not (green before inhaler use; red once dose taken). Alarm stops after dose is taken or after 60 min. An electronic covert adherence log is included to record medication use, which can be uploaded to the study centre.	Fluticasone propionate 250 micrograms twice daily via the Smartinhaler device with covert adherence monitoring and an audiovisual reminder function (twice daily alarms)	Fluticasone propionate 250 micrograms twice daily via the Smartinhaler device with covert adherence monitoring
Christensen et al.	2010	Included by physicians in private practice or hospital ambulatories across Poland	Hypertension	784 patients aged ≥ 18 years	1 year (6 months with either the device or standard therapy, then crossed over to other arm for 6 months)	398/784 in final analysis	Helping Hand Data Capture device. Device has tablet blister cards and has an audiovisual reminder to remind patients once daily to take their medication. It records compliance by recording the date/time of each blister card removal.	Patients received medical treatment with telmisartan once daily (40 or 80mg) and received electronic compliance monitoring with the audiovisual reminder	Patients received medical treatment with telmisartan once daily (40 or 80mg) without the electronic monitor (standard therapy)
de Bruin et al.	2010	HIV outpatient clinic of the Academic Medical Centre in Amsterdam, The Netherlands	HIV	133 patients ≥ 18 years	9 months (2 months baseline measurement, 3 months intervention, 4 months follow-up)	116/133	Medication Event Monitoring System (MEMS) caps—electronic caps that fit on standard pill bottles and register the date/time of each pill bottle opening. Data can be downloaded and printed from the MEMS cap to provide a detailed but comprehensible overview of medication-taking behaviour. The MEMS-view cap used to feedback data to patients as it has a display on top.	Theory- and evidence-based behaviour change intervention—Adherence intervention (AIMS). HIV nurses delivered adherence strategies based on their adherence; those scoring >95% at baseline received "adherence sustaining" intervention; those <95% received "adherence improving" intervention. The "adherence sustaining" involved feedback of MEMS reports, reinforcement, brief discussion of any difficulties. The "adherence improving" included adherence information verbally and using graphs, discussion of patients’ MEMS-reports and comparing this to desired adherence as a motivation for change. Causes of nonadherent events discussed, tailored solutions identified, and patients asked to self-monitor adherence using the MEMS-view cap. At the next visit, patient difficulties discussed, MEMS reports examined, action plans adapted.	No intervention—only usual care consisting of verbal and written information about treatment and consequences of nonadherence; tailoring of the medication regimen and intake schedule to the patients’ daily life; monitoring of side effects; promotion of use of adherence assisting devices; discussion of adherence problems and solutions; feedback about viral load and CD4 count. The MEMS reports were discussed at the end of the study with the control group and intervention delivered if adherence was suboptimal.
De Geest et al.	2006	The University Hospital Basel, or Cantonal Hospital, Aarau, Switzerland	Renal transplant	18 patients ≥ 18 years	9 months (3 months intervention, 6 months follow-up)	13/18	Electronic bottle cap that registers data/time of bottle opening; data downloaded to a computer which generates lists and graphics of medication-taking habits.	Usual care plus 1 home visit after study inclusion, 3 follow-up phone calls at the end of each month. EM printouts were sent to patients before each phone session to enable discussions. Intervention involved behavioural, educational and social support interventions aimed to increase patient self-efficacy in taking medications consistently. Electronic monitoring printouts used for problem detection, proxy goal setting, regular targeted feedback. Interventions were made based on the assessment of reasons for non-adherence identified from the home visit or after discussion with patients about the EAM data. Possible solutions were identified with the patient, nurse and family. Improvements in adherence were rewarded; non-adherence addressed with adherence strategies.	Usual care but physicians were notified if patients were non-adherent; and depression scores suggested moderate/ severe depression or suicidal ideation. Any interventions made by physicians in response to non-adherence were noted.
Dobbels et al.	2017	University Hospitals of Leuven, Belgium	Heart, liver and lung transplant	205 patients ≥ 18 years	6 months (plus 3 month run-in phase)	149/205	Helping Hand Data Capture device. Device has tablet blister cards and a reminder to remind patients to take their medication. It records compliance by recording the date / time of each blister card removal.	Theory-based multicomponent staged tailored medication adherence intervention—with intervention manual, algorithm and scripts to highlight which behaviour change techniques to apply. Multicomponent tailored behavioural interventions (visits2–4) building on social cognitive theory and trans-theoretical model (e.g. electronic monitoring feedback, motivational interviewing).	Usual care—asked to use Helping hand throughout study and complete all visits to control for attention bias (research talked about non-medication topics for 20–30 mins)
Duncan et al.	2013	Rural, university-based hospital in the North-Eastern United States and an urban-based children’s hospital in the Midwest	Asthma	55 participants aged 9 to 15 years	4 sessions of treatment over 2 months with a 3-month follow-up. Total time was 6 sessions (recruitment; 2,4,6,8 weeks; follow-up) across ~5 months	48/55	MDILog-II electronic recording device—captures date/time of inhaler dispensing and whether the participant had inhaled the medication. Attaches to the ICS canister.	**Teamwork intervention**. Importance of parents and youth sharing responsibility for asthma management emphasized and learning methods for addressing conflicts. Families trained in a standardized level of parental supervision of medication use. As youth improved with adherence and reaching adherence goals, supervision reduced based on EM adherence information. **Asthma Education** arm served as an attention control condition—where they received similar education from therapists but did not have access to adherence data from the MDILog-II and parental involvement was not graded based on adherence information.	Standard care; on completion of follow-up, families were provided feedback on child’s adherence and offered an opportunity to receive either of the 2 interventions.
Elixhauser et al.	1990	Outpatient psychiatric clinic of the St. Louis Veterans Administration Medical Centre	Bipolar affective disorder	93 enrolled adult patients	4–8 months depending on frequency of visits set by the patient’s provider (visits could be 2- or 4-monthly)	67/93	Electronic medication monitor for use with oral medication. Involves two plastic blister sheets, each containing 21 blisters holding patient’s medication. The position of the blisters is updated every 15 minutes; if a blister is opened, the time is recorded. Data can be collected with a microcomputer. Printouts provide data on the timing of blister openings with a resolution of 15 minutes.	Monitoring of medication taking only between visit 1 and 2, then feedback of adherence based on electronic monitoring and lithium levels at visit 2, then follow-up at last visit 3. If non-compliance was evident, information was provided about approaches to improve medication taking. Medication received in the medication monitor.	Standard care (no monitoring) between visit 1 and 2, then feedback of adherence based on lithium levels alone at visit 2, then follow-up at last visit 3. Discussions about reasons for out-of-range values and suggestions for improving compliance provided. Medication received in a usual cylindrical vial.
Erickson et al.	2005	Hypertension specialty outpatient clinic within a large university-affiliated medical centre	Hypertension	42 subjects aged ≥ 21 years	3 months	37/42	Medication management system (MMS). The MMS uses patient-specific information to tailor the interactive technology to each patient, and aims to enhance adherence and communication between the patient and health provider. It includes the MedManager device which stores medication, provides reminder signals through an audio alarm and visual text message to alert patients to take a dose or enter data, and collects date/ time of opening of a medication well, blood pressure readings and potential symptoms of adverse effects of medication. Data are transmitted nightly to the central computer. Reports of patient medication use and clinical parameters were generated monthly and sent to the patient and physician.	Medication management system with standard medical care. This MMS was customized for the patient’s medication regimen, daily activities and any special instructions for administration based on patient, pharmacist and physician review.	Standard medical care alone
Forni Ogna et al.	2016	Cardiology intervention unit of the Lausanne university hospital	PCI with stent	123 adults	6 months	117/123	MEMS cap	Standard of care (SOC) + adherence electronic monitoring (EAM) group, in which drug intake was recorded but kept blinded until the study end, versus another intervention group—an integrated care group, with regular feedback on recorded adherence. Integrated care group = downloaded data every 6/52 and fed back at follow up in semi-structured motivational interviews with nurse, or pharmacist, and patients. SOC+EAM—recorded data electronically but patient and study staff blinded until study end.	Standard care
Foster et al.	2014	General practices in Greater Sydney, Australia	Asthma	143 patients aged 14 to 65 years with moderate–severe asthma	6 months with just 2 study visits (enrolment, then follow-up) with telephone data collection at BL, 2,4 and 6 months	129/143	SmartTrack EAM with audiovisual reminder function for use with preventive medication. Records date, time and number of actuations used and uploads data monthly to a secure website. Asks 3 onscreen questions about asthma control each month.	Twice daily customizable audiovisual inhaler reminders and feedback (IRF) delivered by GPs vs personalized adherence discussions between the GP and patient about adherence (PADS) vs IRF + PADs. PAD involved a short questionnaire about barriers to inhaler use with a discussion about the key barriers with goal setting and achievement strategies	Usual care based on "Asthma Cycle of Care" including one-off checking and teaching of inhaler technique and writing asthma action plans. Patients were offered a record of their adherence after the study.
Frick et al.	2001	Sexually Transmitted Disease and Family Planning Clinics, Coast Provincial General Hospital in Mombasa—the government referral hospital for the coastal region of Kenya	HIV model but tested with multivitamins	140 women aged between 18 and 45 years	1 month	120/140 in final analysis	RemindRx®—Microelectronic alarmed medication vial with programmable dosage administration times that records date/time when a button on the vial is depressed. The button also serves to silence the alarm. Data could be downloaded into a computer.	Electronic medication vial with alarm	Electronic medication vial with no alarm
Gregoriano et al.	2019	In- and outpatients from several hospitals in the Basel region	Asthma, COPD	169 adults	6 months	149/169	Smartinhaler and Polymedication Electronic Monitoring System (POEMS)	Acoustic reminder for inhalation and receives support calls when the medication is not taken as prescribed;—The reminder was automatically set at the time, when the patients had to inhale their dose (every day) and not only when a dose was missed. The support calls occurred only if the patients had not inhaled their medication as prescribed for more than 2 consecutive days. Recorded data with the Smartinhalers were uploaded daily at 00:00 to a web-based database via a wireless connection, so the health provider was able to control daily the performed inhalations and to intervene when necessary, by controlling the data on the database.	No reminder nor additional assistance or feedback regarding their medication adherence behaviour.
Hardstaff et al.	2003	Renal and Liver Transplant Unit, Freeman Hospital, Newcastle-upon-Tyne, UK	Renal transplant	75 renal transplant adult patients	12 months	48/75	Smart Top bottle. These bottles have specialised lids containing a microprocessor that records date/time of bottle opening/closing. Information downloadable into a computer database.	Received adherence feedback at first outpatient clinic appointment but then no further feedback	Received no feedback throughout the trial
Henriksson et al.	2016	Karolinska University hospital in Stockholm, Sweden	Renal transplant	80 adults	12 months	74/80	EAM with cellular capabilities (tracking device via Global System for Mobile Communications)	The patients loaded the EAM with a week’s worth of medication at a time. At the prescribed time for taking the medication, the EAM gave visual and audible signals. If the patient did not take their medication, the audible signal was repeated with increasing frequency for 120 minutes. After this (or after the medication was taken), the EAM sent an SMS message to the web-based software, thus providing information about patient compliance.	Standard care, no EAM
Hermann et al.	2011	Glaucoma clinic at the University Hospital in Athens, Greece	Glaucoma	37 patients ≥ 18 years with glaucoma or ocular hypertension	4 weeks	36/37	EAM for use with brimonidine eye drops 0.2%. Records time/date of use by measuring bottle motion and squeezing. Device not able to be separated from the bottle.	Open adherence monitoring with brimonidine twice (BD) or three times daily (TDS)	Masked adherence monitoring with brimonidine twice or three times daily
Joost et al.	2014	Erlangen University Hospital, Germany	Renal transplant	74 renal transplant patients ≥ 18 years	1 year	67/74	Medication Event Monitory System (MEMS) caps	Same as control plus an intensified pharmaceutical care programme targeting daily drug adherence in the year after transplant. This included additional inpatient and outpatient pharmaceutical care and counselling and a structured adherence management module focusing on adherence support. This was delivered during days 6–20 post-transplant (3 x ~30 min sessions), then consultations with the clinical pharmacist with oral & graphical feedback on adherence data occurred after discharge at least once per quarter up to a maximum of once monthly in the year post-transplant	Standardized drug and transplant training including 15-page written information on medications, rejections, tumour risks, infections and a 1 hour training session from the transplant physician on medications, and another 1 hour from nurses regarding practical application of their medication. Follow-up visits with the transplant centre continued as per usual, but there was no additional contact with the clinical pharmacist beyond regular MEMS refill and adherence data collection.
Kozuki et al.	2006	Community mental health centres in the Pacific North-west	Psychotic disorders	30 adult patients	3 months	28/30	eDEM—electronic monitoring cap that records daily execution of the regimen and produces a chronology of the time the medication was taken each day. Information can be downloaded and presented on a computer screen.	Visual-feedback therapy with structured psychodynamic therapy and visual feedback via monitoring cap to increase insight about medication behaviours and work on affect dimensions. Delivered every 2 weeks for 3 months. Focus is on both behaviours and emotional needs of persons with psychotic disorders and has both a behavioural (insight into pill-taking and acceptance of medications with aim to improve affective reactions) and psychodynamic component (encouraged to express concerns about medications / illness/ issues). Information from the cap was shown to patients on a computer screen at each session, then related this to patients’ appraisals of the behaviours.	Supportive counselling group—attentive listening only therapy technique used. Issues related to the medications not discussed. Delivered every 2 weeks for 3 months for 20–30 min per session. Used to control for confounding of attention from therapists.
Matteson-Kome et al.	2014	Mid-western outpatient Inflammatory bowel disease (IBD) clinic	IBD	6 adults ≥ 18 years	3 months intervention phase with a 60-day screening phase to identify non-adherent patients (< 85%) = total 5 months	5/6	Medication Event Monitoring System (MEMS Track Cap -) electronic bottle cap that monitors dosing (not timing) of medication.	Continuous self-improvement intervention (CSI) involving data evaluation and system refinement to help change behaviour by focusing on the patients’ personal systems rather than on their motivation/ intention. It fosters ritualistic and habitual health behaviours and requires less effort, motivation, and intention to maintain changes. This involved a face to face intervention assessing MEMS data after education on brief personal system theory. Patterns of adherence were analysed from MEMS data for patterns of non-adherence and potential personal system changes discussed for patients to implement during the 3-month study	Attention control intervention—face to face educational session presenting information on IBD education topics such as medical therapy, side effects, extra-intestinal manifestations of IBD and surgical modalities. MEMS also received.
McKenney et al	1992	Residence in a retirement community or attending a primary care centre, Virginia	Hypertension	70 ambulant patients ≥ 50 years	2 x 12-week phases	Phase I: 69/70 Phase II: 59/70	Prescript TimeCap—an electronic compliance aid consisting of a medication vial with a cap displaying the last time the cap was removed.	Timepiece cap alone (vs control) for Phase I of study; then timepiece cap alone vs cap + cards for recording BP readings at each clinic visit vs cap + BP recording cards + home BP monitoring and documentation in the cards	Standard medication vial
Mehta et al.	2019	University of Pennsylvania general internal medicine practices	Hypertension	151 adults aged 18 to 75 years	4 months	126/151	Electronic pill bottle (arm 1) and bidirectional text messages (arm 2)	Arm 1: electronic pill bottle (AdhereTech): electronically monitor openings and transmit them to online platform. Participants received one of two daily feedback messages, depending on their adherence the day prior. Arm 2: bidirectional texting arm received text messages via the online platform, prompting the participant to reply via text with his/her adherence for that day. Mirroring the pill bottle arm, the subsequent days the feedback.	Usual care provided by clinical practice
Morton et al.	2017	Hospital clinics in Sheffield or Rotherham (UK)	Asthma	90 children aged 6 to 16 years	12 months	79/90	Smartinhalers’ and ‘Smartturbos’	Smartinhalers’ and ‘Smartturbos’ that delivered reminders when forgotten and was upload to clinician and discussed at 3 month check up	Same smart inhaler but no alarms and no review by clinician
Murray et al.	2007	University-affiliated, inner-city, ambulatory care practice—general medicine and cardiology practices of Wishard Health Services, Indianapolis, Indiana and Wishard Memorial Hospital	CHF	314 low-income patients ≥ 50 years with heart failure	12 months (9-month multi-level intervention with 3-month poststudy phase)	270/314	Medication Event Monitoring System (MEMS) V prescription container lids that recorded the time/date of each opening and closing. The MEMS cap was labelled with the same icon as the container body to allow correct matching of medicines.	Pharmacist intervention to improve adherence and health outcomes. Involved a baseline medication history, assessment of patient medication knowledge and skills, patient-centred verbal instructions and written materials about the medications, icon-based labelling of medications and a timeline to remind patients when to take their medications. The pharmacist monitored medication use, body weight, healthcare encounters and fed back information as needed to other health professionals.	Usual care which did not include patient-centred materials or any further contact with the intervention pharmacist besides an initial medication history
Nides et al.	1993	University of California at Los Angeles and John Hopkins University	COPD	251 patients aged 35 to 60 years	4 months	205/251	Nebulizer Chronolog—a microprocessor device recording time and date of actuation that can be downloaded into an IBM-compatible computer.	Patients informed about the function of the device and received printed copies of their EAM record of inhaler use at end of weeks 1 and 7 of the 12-week smoking cessation program. The health educator and participant jointly reviewed the pattern of inhaler use—praise given if usage satisfactory, and behavioural strategies developed for problem areas. These feedback sessions continued at each 4-month follow-up visit	Patients only told the device recorded amount of inhaled drug use—no information given that it was able to record patterns of use. No feedback given.
Okeke et al.	2009	Glaucoma services of the Scheie or Wilmer Eye Institutes	Glaucoma	66 patients ≥ 18 years	6 months (initial 3-month observational period of which 2 months of data from week 2 to 10 were used, plus 3-month intervention period)	Not stated	Dosing aid bottle—squeezes the drop from the bottle and records the time and date of delivery.	Educational video stressing importance of adherence, rationale, effects, and how to maximize adherence, a structured review of current barriers to drop taking and discussion of possible solutions with a study coordinator. Regular phone call reminders discussing administration, side effects, difficulties with drops—weekly for first follow-up month then every other week for next 2 months; plus audible and visible reminders on the dosing aid device used	No additional intervention beyond being told it is important to take your eyedrops as prescribed.
Onyirimba et al.	2003	Asthma Centre at Saint Francis Hospital and Medical Centre	Asthma	30 adult patients	10 weeks	19/30	MDI Chronologs	Standard asthma care plus direct, non-judgmental clinician-to-patient feedback discussion on their inhaled steroid and beta-agonist use (date/time of use) on all visits using electronic print-outs. This was fed back at days 7, 14, 21 and 42.	Standard asthma care including asthma education and development of a management plan (BL, days 7, 14, 21 and 42). Actuation data blinded to patient, clinician and other caregivers
Reddy et al.	2016	Medical centre in Philadelphia, US	CAD	125 veterans with known CAD and poor adherence, aged 30 to 75	13 weeks	117/126	"GlowCap. The bottle has a computer chip in the lid that communicates with a cellular connected plug-in nightlight. When all features are activated, the GlowCap monitor changes colour 1 h before the scheduled time to take the medication. If the medication is taken during this period, the pill bottle does not sound an alarm. If the medication is not taken within the designated period, the bottle flashes and sounds an alarm"	The individual feedback participants received a bottle with a daily alarm and a weekly adherence feedback report. Weekly feedback reports displayed participants’ medication adherence and assigned a value for weekly performance based on the number of days that they had opened the bottle. Participants in the partner feedback also had a copy of the report sent to their designated family member, friend, or peer. All participants and partners were trained on the interpretation of the weekly adherence report.	All patients received educational material on the importance of adherence to statin medication. The control group received this device, but none of the patient features were activated (no alarm or notification).
Rigsby et al.	2000	Department of Veterans Affairs HIV clinic and the University of Connecticut Infectious Diseases Study Center—a community-based HIV clinical trials site in the City of Hartford Health Department in Hartford, Connecticut	HIV	55 HIV-infected adult subjects	12 weeks (intervention 0–4 weeks, then follow-up at weeks 8 and 12).	46/55	MEMS caps—fixed to the medication with the lowest baseline adherence in the 1-week baseline period.	Weekly sessions for four weeks of cue dose training with MEMS feedback (CD), or cue dose training with cash reinforcement for correct bottle openings (CD + CR). Cue-dose training linked medication taking to daily habits as cues and used MEMS data to reveal missed doses and suggest alternative cues. Contingency reinforcement using graduated cash payments at each weekly meeting for 4 weeks based on consecutive correctly timed bottle openings formed the base of the CD + CR intervention. The reinforcement began at $2 per correct dose and increased with each consecutive correct dose to a maximum of $10 per day. If the dose was not taken on time, it reset to $2.	Non-directive inquiries about adherence—asked about adherence in the week preceding the visit and encouraged to improve adherence. MEMS data not fed back.
Rosen et al.	2004	Primary care clinic at the VA Connecticut Healthcare System	Diabetes	79 adult patients enrolled but only 33 had lower than 80% baseline adherence and were randomised	4 months intervention + 3 months follow-up (no intervention, assessment only)	33/33	MEMS caps/Smart Caps	Cue-dose training with Smart Caps that display the number of hours since last bottle opening—programmable to beep at pre-determined times. Patients instructed to consider cues to remind them to take the medication with opportunities to discuss barriers to adherence. MEMS data given to health providers each month—and if patients had scheduled appointments, the MEMS data would be discussed with the patients.	Supportive counselling for first 5 patients based on self-reported (not MEMS) data but the supportive counselling had elements of the active intervention as the same people gave the counselling, so the next 12 patients had assessments only with no active counselling and no presentation of MEMS data to providers
Rosen et al.	2007	HIV clinics in the greater Hartford, Connecticut area	HIV	56 adult participants	32 weeks	36/56	MEMS caps with downloaded data to a computer. Print out shows date and time of each bottle opening over the preceding weeks and the list of doses taken.	Weekly contingency management-based counselling for 16 weeks then 16 weeks of additional data collection. At counselling, data from the MEMS caps were reviewed with patients to identify circumstances surrounding missed doses and identified cues to remind them to take the dose. Responses to the medications, routines for medication taking and efforts to cope with HIV also reviewed. Brief substance abuse counselling conducted. Participants were reinforced for MEMS measured adherence (within 3 hours of agreed times for dosing) with drawings from a bowl for prizes and bonus drawings for consecutive days of perfect adherence, and for consecutive weeks. There was a 26.7% chance to earn per $1.00 card, a 7.6% chance for $20.00, and a 0.2% chance of earning $100.00. Potential total earnings averaged $800. The bonus draws reset if perfect adherence did not occur. In order to be certain that participants sampled the reinforcement, participants received two draws for attending each of the first two counselling sessions. In addition, for the first two weeks, participants were reinforced for having taken any doses on the designated day, whereas afterwards, reinforcement was only provided when all a day’s doses had been taken on time. The providers all received monthly letters of the proportion of doses taken from the MEMS throughout the 32 weeks, but this was not actively followed up with the provider.	Weekly supportive counselling for 16 weeks as the "attention control" condition. Participants were asked about their adherence and offered support for efforts to improve adherence. MEMS data was not reviewed with the participants though and urine toxicology testing not conducted. Only an initial review of substance abuse was done and referrals made for treatment. Monthly letters on adherence (self-reported not from MEMS) were sent to providers
Ruppar	2010	Senior centres, senior living facilities, churches in two Midwestern US cities	Hypertension	15 subjects aged 60 years or older	28 weeks (8-week run-in period + 8-week intervention + 12-week follow-up)	15/15	MEMS electronic medication bottle cap with a digital display that provided daily adherence feedback of date and time of opening of the bottle during the 8-week intervention.	Behavioural feedback intervention with biweekly medication adherence and BP feedback (participants were informed of their adherence rate since the last visit and were shown a graphical display of their adherence behaviour to date; degree of change in the participants’ BP discussed and how it could have been impacted by improvements in adherence), habit counselling, review of medication-taking skills, medication and disease education, medication instruction card.	Received no adherence feedback and was seen by the investigator at weeks 12 and 20 only. Educational materials on managing arthritis pain were provided.
Russell et al.	2011	Tertiary care transplant centre located in the Midwestern United States	Renal transplant	15 adult renal transplant recipients aged 21 years or older	9 months (3-month screening phase + 6- month intervention)	15/15	MEMS Track cap—date and time of removal of the cap from the vial,	Continuous self-improvement intervention (CSI) involving collaboration between the participant and clinical nurse specialist on identification of the person’s life routines, important people, possible solutions to enhance medication taking. Monthly medication taking feedback was also delivered via a graphic printout of daily adherence from electronic monitoring. This was conducted monthly during the 6-month intervention.	Attention control—provided with educational brochures from the International Transplant Nurses Society to address healthy post-transplant behaviours. The first brochure was delivered via a home visit and subsequent brochures were mailed. Monthly phone calls to review the brochures and ask participants if they had any questions about the information were made to provide equal attention time and perceived benefits to the control group.
Sabin et al.	2010	Dali Second People’s Hospital in Dali, Yunnan province, China	HIV	80 enrolled, 68 subjects ≥ 18 years old randomised	12 months (pre-intervention phase months 1–6 of monitoring to identify high or low adherence for stratification; intervention period months 7–12)	64/68	Med-ic—Electronic drug monitor pill bottle.	Counselling with feedback from electronic drug monitors. Data from the monitor downloaded each month and the previous month’s data was reviewed with the patient. Those with less than 9% adherence were flagged for counselling with a physician or nurse using the monitor adherence data after the clinic. Data was provided to both the patient and the clinician as a printout with the percent of doses taken, percent taken on time, and a visual display of doses taken by time. Reasons for missing or off-time doses were discussed and problems/challenges identified at the counselling.	Standard care with no feedback of collected adherence data to the patient nor the clinician. However—those whose monthly written self-reports indicated < 95% adherence also received further counselling. The main difference with the control is that the ’flagging’ for counselling relies on self-report rather than electronic drug monitor data.
Smith et al.	2003	Hospital-based infection disease clinic at the University of North Carolina Hospitals in Chapel Hill, NC	HIV	43 individuals ≥ 18 years	3 months but clinical outcomes assessed within 1 year of randomisation	Not stated	MEMS electronic monitors on medication bottles.	Self-management intervention based on feedback of adherence performance and principles of social cognitive theory and self-regulation—3 components of information exchange, skills development and social support enlistment. The program included medication education as per the control group, skills training and development exercises, monthly visits for medication consultations and one-on-one counselling for 3 months, monthly feedback of adherence performance using diary notes, supportive feedback about how closely they adherence to the dosing schedule and with graphical dosing information from the electronic monitoring caps. Goal setting was also done.	Usual care with medication education—written and verbal—and assistance with scheduling of doses. Strategies to improve adherence were discussed. No follow-up visits
Sulaiman et al.	2018	University specialist asthma hospital clinic	Asthma	218 adults with stage 3 to 5 asthma	3 months	148/218	INhaler Compliance Assessment (INCA) attached to inhaler to make a digital audio recording each time the inhaler is used	Basing on information obtained directly from the INCA acoustic recording device, the group discusses patterns of adherence and training on technique of inhaler use as part of biofeedback-guided training.	Generalised strategies to improve adherence, while technique errors are corrected using checklists. Repeated training in inhaler use, adherence and disease management, no biofeedback
Sutton et al.	2014	Primary care clinics in Oxfordshire, Buckinghamshire, Suffolk, Essex, Huntingdonshire	Diabetes	226 adults ≥ 18 years	8 weeks	184/226 for adherence analysis; 193/226 for HbA1c analysis	TrackCap	Electronic container for medication	Standard medication packaging
Tashkin et al.	1991	John Hopkins University and UCLA	COPD	237 adults of a larger intervention group that received a group-based smoking cessation programme, education, counselling and NRT	12 weeks (4 months)	197/237 (40 forgot devices, missed the appointment or had malfunctioning devices)	Nebulizer Chronolog—small, portable electronic device housing a pMDI—records the date and time of each actuation and is read out by an IBM PC.	Informed of the function of the Chronolog and given feedback of the adherence information to enhance adherence. For the feedback participants, the Chronolog memory was read by the interventionist on several occasions over the 12-week program. If the feedback participants were not using the bronchodilator three times per day at appropriately spaced intervals or were not using 2 actuations per set, the information was given to them and the interventionist worked with them to improve adherence to the prescribed regimen. Those who had good compliance were congratulated and encouraged to continue proper inhaler use.	Blinded to adherence monitoring function of the device.
van Onzenoort et al.	2012	Maastricht University Hospital, Maastricht, The Netherlands and surrounding general practitioners’ practices	Hypertension	470 patients ≥18 years with mild–moderate hypertension as part of a larger HOMERUS trial	1 year with seven follow-up visits (a placebo run-in period of 4 weeks was also conducted before study initiation)	Not stated	MEMS cap V TrackCaps	Adherence monitoring with MEMS (but adherence was not fed back) and pill count	Adherence monitoring by pill count alone
Vasbinder et al.	2017	Outpatient clinics in the Netherlands	Asthma	219 children aged 4 to 11 years	12 months	213/219 (only analysed 209/219)	Real-time medication monitoring (RTMM) device, which was connected to the pressurised metered-dose inhaler (pMDI) and recorded the time and date of administered ICS doses. Immediately after each actuation of the pMDI, data were sent to the study database through the mobile telephone network	The intervention group received tailored SMS reminders, sent only when a dose was at risk of omission	No SMS reminders but still had RTMM device that recorded time and date of ICS dose
Velligan et al	2013	Community mental health centre from two counties in Texas	Schizophrenia	142 patients aged between 18 and 60	9 months after 1-month baseline assessment of adherence monitoring with the MM and pill count	132/142	Med-eMonitor (MM)—an electronic medication monitor that prompts use of medication, cues medication taking, warns patients when the wrong medication is taken or at the wrong time, records complaints and alerts staff of failures to take medication as prescribed.	PharmCAT (in-person)—supports medication taking with an array or environmental supports e.g. using pill containers, signs, alarms, checklists established in weekly home visits from a PharmCAT therapist vs Med-eMonitor (electronic) adherence intervention which is the only support and only contact is via phone if patient missed doses (adherence server checked every 3 days); phone contact addressed practical issues, or motivation issues. Both aim to bypass controlled processes in favour of automatic processes and habit formation—reinforcing adherence with electronic messages or social reinforcement.	Treatment as usual—case management and psychiatry appointments at the community mental health center
Wilson et al.	2010	Two academic medical centres, a community health centre, general medicine practice and private infectious diseases practice in the US	HIV	156 adult patients	6 study visits (BL, visits 1–4 before a provider visit, final (6–12 weeks after 4th provider visit)	106/156	MEMS cap	Cross over study. Three-page report of MEMS adherence data given to physician prior to a routine office visit. Self-report data on adherence, patients’ beliefs about therapy, reasons for missed doses, alcohol and drug use and depression also given to the physician. Group 1—received the report prior to the 2 consecutive visits followed by 2 visits with no report. Group 2—no report for first 2 visits, then report with the last 2 visits. The first and third visits were recorded (one intervention, one control)	
Wu et al.	2006	John Hopkins Moore (HIV) clinic	HIV	64 patients ≥ 18 years	6 months	48/64	Disease Management Assistance System (DMAS)—a prompting device that verbally reminds patients at medication times and records doses when manually pushed; eDEM to measure adherence.	DMAS + monthly 30 minute adherence educational session	Education only
Yeh et al.	2017	Paediatric MS specialist hospital clinic, US	MS	71 children aged 10 to 18	6 months	49/71	MEMS cap + behavioural feedback	Subjects received a supplemental device which downloaded Adherence data from the MEMS cap for use by the behavioural interventionist during a telephone feedback session at 1,2 and 3 months post-enrolment. Phone called based on MI based on adherence (parents not involved)	Video related to the disease modifying therapy (DMT) in paediatric MS sent at 1, 2 and 3 months

HIV, Human Immunodeficiency Virus; HAART, Highly active antiretroviral combination therapy; DMAS, Disease Management Assistance System; CHF, Congestive Heart Failure; T2DM, Type 2 Diabetes Mellitus; pMDI, pressurised metered dose inhaler; mAMS, Medication adherence management system; EAM, Electronic medication monitor; NZ, New Zealand; MEMS, Medication Event Monitoring System; MMS, Medication Management System; SOC, Standard of Care; IRF, Inhaler reminders and feedback; PAD, Personalised adherence discussion; COPD, Chronic Obstructive Pulmonary Disease; POEMS, Polymedication Electronic Monitoring System; UK, United Kingdom; BD, twice daily; TDS, three times daily; CSI, Continuous self-improvement intervention; IBD, inflammatory bowel disease; CAD, coronary artery disease; CD, cue-dose training; CR, cue-dose training with reinforcement; INCA, INhaler Compliance Assessment; ICS, inhaled corticosteroids; SMS, short message service; RTMM, real-time medication monitoring system; MS, multiple sclerosis; DMT, disease modifying therapy.

The most common EAM device type was an electronic cap fitted onto an oral medication bottle (the ‘Medication Event Monitoring System (MEMS)‘ (n = 14, 30%) [26, 37, 39, 40, 42, 43, 47, 48, 50–55] or similar (n = 10, 21%) [38, 41, 46, 49, 56–61]. The four (9%) other studies of oral medicines used electronic medication blister cards [14, 62–64]. Some used EAM devices that fitted to a specific medication formulation such as inhalers (n = 12, 26%) [11, 27–35, 65, 66] or eyedrops (n = 2, 4%) [67, 68]. Five (11%) [36, 44, 45, 69, 70] used an integrated medication management system (MMS) which included recording of dosing times and symptoms, reminders about lifestyle and /or medication-taking, and information about disease control.

Most studies, except two early studies [46, 63], used electronic monitoring to measure adherence, though this was frequently used with other measures such as serum medication levels [39, 40, 59, 63], self-report [14, 38, 39, 41, 43, 49, 51–55, 64–66, 71], adherence questionnaire [36, 45, 51, 61], pill count [38, 48, 51, 59, 69, 70], canister weight [65, 66],or prescription refill data [52, 55].

Of the 47 included articles, 27 (57%) studies provided sufficient data for the primary outcome meta-analysis, through the published manuscript or author contact. Fourteen (30%) authors were uncontactable, three (6%) studies did not report on adherence differences in both control and intervention groups and two (4%) authors could not provide further data. From these 27 included studies, 25 were eligible for the secondary outcome analysis of studies measuring taking adherence, and 24 of studies using objective adherence measures.

### Effect of EAM on medication adherence

**Fig 2:**
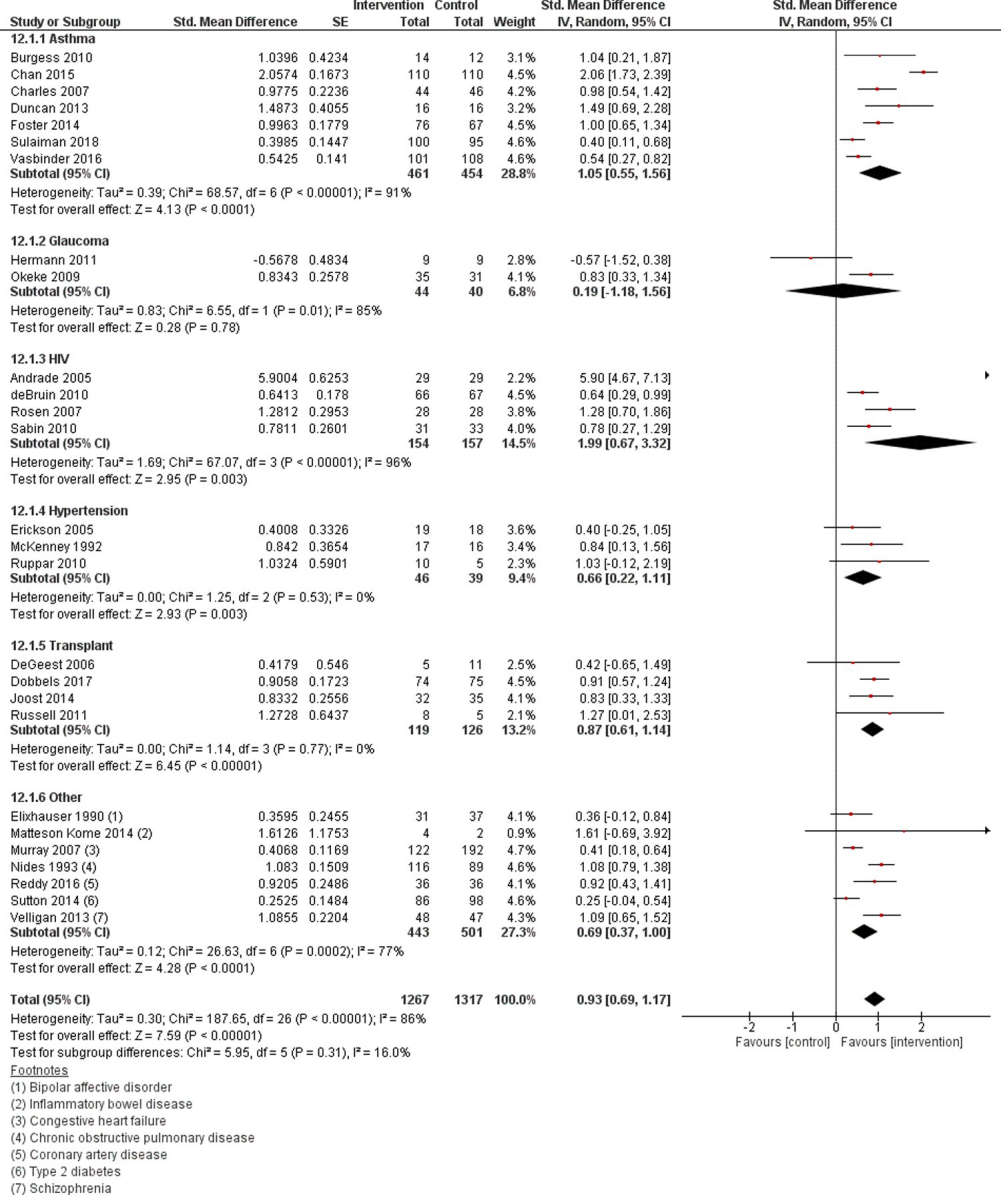
Forest plot of effect of the electronic adherence monitoring intervention compared to control on medication adherence for studies with available data (n = 27) by chronic condition. SE, standard error; CI, confidence intervals for effect size.

The primary outcome analysis of pooled data from 27 studies (n = 2584) showed that the intervention group (n = 1267) had significantly better adherence than control (n = 1317), (MD = 0.93, CI: 0.69 to 1.17, p = <0.0001). Statistically significant heterogeneity was present (Q = 187.65, p = <0.0001) and of substantial degree (I^2^ = 86%). The forest plot for all studies is shown in Fig 2.

The secondary outcome analysis of the 25 studies (n = 1127 in the intervention, n = 1175 in the control) that measured taking adherence showed a positive effect size (MD = 0.95, CI: 0.69 to 1.22, p = <0.0001). Simlarly, analysis of the 24 studies using objective measures of adherence (n = 1131 intervention, n = 1164 control group) also showed a statistically significant positive effect size (MD = 1.02, CI: 0.76 to 1.28, p = <0.0001).

#### Study-level characteristics: Subgroup analysis

Separate subgroup analyses are shown in Table 2. All subgroups had positive effect sizes, with no significant differences among subgroups except for the “complexity of intervention” variable (p = 0.01). EAM-only interventions did not improve adherence (SMD = 0.24, CI:-0.35 to 0.84) as much compared to interventions where the EAM was used with a reminder and / or health professional input (SMD ranged from 0.73 (EAM + health professional input) to 1.51 (EAM + EAM reminder) (CI range: -0.54–2.22).

**Table 2:** Effect of study-level characteristics on medication adherence (n = 27).

Variable	Subgroups	No. of studies	No. of participants	SMD	CI	p-value for interaction*
**Age**	Children (<18 years)	4	467	1.28	0.36 to 2.2	0.37
	Adults (≥18 years)	23	2097	0.85	0.62 to 1.02	
**Healthcare setting**	Primary / ambulatory care /outpatient	20	2023	0.85	0.59 to 1.10	0.32
	hospital setting	7	561	1.14	0.63 to 1.64	
**Complexity of the intervention**	EAM only	3	235	0.24	-0.35 to 0.84	**0.01**
	EAM + EAM reminder	7	829	1.51	0.81 to 2.22	
	EAM + health professional input	13	1288	0.73	0.54 to 0.93	
	EAM + EAM reminder + health professional input	4	232	1.05	0.77 to 1.33	
**Method of adherence feedback** ^a^	Direct-to-patient	6	655	0.96	0.37 to 1.54	0.15
	Healthcare provider facilitated	12	1062	0.79	0.54 to 1.03	
	Both	7	665	1.42	0.82 to 2.01	
**Timing of adherence feedback**	Real time (immediate)	9	1048	1.03	0.66 to 1.40	0.53
	Delayed	18	1536	0.88	0.57 to 1.19	
**Study duration**	Short (6 months)	11	856	0.70	0.40 to 0.99	0.09
	Long (over 6 months)	16	1728	1.08	0.74 to 1.43	
**Blinding** ^b^	Blinded to adherence monitoring function	7	741	1.06	0.59 to 1.53	0.09
	No blinding	10	982	0.62	0.46 to 0.79	

SMD, standardised mean difference; CI, confidence intervals for effect size; EAM, Electronic medication monitor

^a^Two studies did not provide adherence feedback to participants and were not included in the subgroup analysis.

^b^It was not clear in 10 studies whether participants were blinded to adherence monitoring. These were excluded from the subgroup analysis.

### Effect on clinical outcomes

Table 3 shows the effects of EAM on clinical outcomes across the 47 included studies summarised narratively. Nine studies (19%) did not assess clinical effect [26, 38, 54–57, 65, 66, 68]–most these (7/9) were of a shorter study duration (six months or less). There were 38 (81%) studies that reported clinical outcomes; ten (26%) reported statistically significant improvements [27, 28, 30, 32, 37, 39, 46, 47, 52, 62].

**Table 3:** Summary of interventions and effect on clinical outcomes (n = 47).

Author	Condition	Participants blinded to adherence monitoring	Type of EAM	Method of adherence feedback (Direct to patient or Indirect via health professional)	Immediate real-time feedback to participant about adherence	Method of adherence measurement (for both groups unless otherwise stated)	Effect on adherence	Size of effect	Clinical effect	Clinical measure	Main Clinical Outcome
Andrade et al., 2005 [36]	HIV	Not stated	Integrated	Both	No	EM, Adherence questionnaire	0, + Mean (±SE) adherence scores did not differ between groups (80 ± 2.3% vs 65 ±2.7%)	+15% (overall), +20% (memory-impaired subgroup)	+/-	Viral load, CD4	At week 24, plasma HIV RNA load was undetectable for 34% in the intervention group and 38% of the control (P = 0.49). Overall: Greater reduction in viral load, no difference in CD4
Artinian et al., 2003 [69]	Congestive Heart Failure (HF)	Not stated	Integrated	Direct	No	EM (intervention only), Pill count (both groups)	Not compared; only reported that the monitor group had 94% adherence	Not available	+/-	HF self-care scale, 6 min walk, Minnesota Living with HF Questionnaire for QOL, NYHA FC	No significant group by time interaction (F1, 16 = 0.02, P = -0.902), suggesting that there was no significant difference in the amount of HF self-care improvement between groups. Overall: No effect on clinical measures but had better physical QOL in the monitor group between baseline and follow-up.
Brath et al., 2013 [62]	Hypertension and dyslipidaemia	Not stated	Simple EAM	Direct	No	EM (intervention phase); pill count (control phase)	0 Medians in both intervention and control groups reported as 1 (1–1 for interquartile range), with no significant differences other than for metformin (p = 0.04)	Not available	++	BP Control, HbA1c, fasting blood glucose conc., blood cholesterol concentration	BP improved from 133/75 to 127/70 (p = 0.02/0.0003) from beginning to end of crossover phase; Total cholesterol improved from median (lower-upper quartile) = 166 (147–183) to 155 (141–167); p = 0.02. Overall: Improved BP, total cholesterol. Nil significant changes in glucose, body weight, or LDL or HDL cholesterol
Burgess et al., 2010 [27]	Asthma	No	Simple EAM	Indirect	No	EM	++ Mean (SD) adherence in intervention 79.0 (13.1)% vs control 57.9 (25.3)%; P<0.01) (data from author).	+21%	++	Reliever use, lung function, exacerbations, asthma symptoms	Intervention = 3 exacerbations vs control = 1 (p = 0.4); change in FEV1 from baseline = 13.8 (intervention) vs 9.8 (control) (p = 0.9); number of people using reliever medication ≥3 times per week = 2 (intervention) vs 0 (control). Overall: Improved asthma control based on changes in FEV1 and number of exacerbations but not significant between groups
Chan et al., 2015 [28]	Asthma	Yes	Simple EAM	Direct	Yes	EM	++ Intervention mean (SD) adherence: 78.5 (18.7)%, control 35.0 (23.2)% (data from author)	+54%	++	Asthma Morbidity Questionnaire, ACT	Mean (SD) change from baseline: Intervention +3.86 (5.8) vs control +2.62 (5.9). Overall: Improved asthma control based on asthma questionnaires
Charles et al., 2007 [29]	Asthma	Yes	Simple EAM	Direct	Yes	EM	++ Intervention mean (SD) adherence: 88 (16)% vs control 66(27)%	+22%	0	ACQ	Change from baseline = 0.5 in both groups; no SD given Overall: No effect on clinical
Christensen et al., 2010 [14]	Hypertension	Not stated	Simple EAM	Direct	No	EM, Self-report	0 Self reported compliance was higher in the intervention group than control (5.5% difference) in the 1st 6 months, but the opposite was seen in the crossover (-2.1%).	+6% then -2% with crossover	0	BP control	BP systolic change at 6 months: intervention group before cross over = -28.8 vs. control group -28.3 mmHg (p = 0.801) BP diastolic change at 6 months: intervention -13.4 vs control -13.6 mmHg (p = 0.808) Overall: No effect on clinical
de Bruin et al., 2010 [37]	HIV	No	Simple EAM	Both	Yes	EM	++, + Effect on timing adherence was significant (*F*(1, 129) = 14.11, *p <* 0.001, mean difference = 7.40% [3.50–11.30%])	+7% (overall), +15% (<95% baseline adherence)	++	Viral load, CD4	Logistic regression showed that the intervention group had a higher chance of being undetectable at postintervention than the control group (p <0.05, OR [95% CI] = 3.32 [1.13–9.80]). Overall: Greater reduction in viral load
De Geest et al., 2006 [56]	Renal transplant	No	Simple EAM	Indirect	No	EM	0 Chance of non-adherence reduced more in the intervention vs control group but difference was not significant (p = 0.31).	Not available	Not measured	-	Not measured
Dobbels et al., 2017 [64]	Heart, liver and lung transplant	No	Simple EAM	Both	Yes	EM, Self-report	0 Intervention group had a 16% higher dosing adherence post-intervention (95.1% intervention vs 79.1% control group; p<0.001), resulting in odds of adherence being 5 times higher in the intervention than in the control (odds ratio 5.17, 95% confidence interval 2.86–9.38).	Not available as reported as OR.	+	Event-free survival	5-year clinical event-free survival was 82.5% (intervention) vs 72.5% (control) relative risk 0.64, 95% CI 0.38–1.08; log-rank test p = 0.18) Overall: Higher rates of event-free survival (trend)
Duncan et al., 2013 [30]	Asthma	Yes	Simple EAM	Indirect	No	EM	++ Teamwork intervention group had significantly higher medication adherence rates—at 20 weeks, mean (SD) adherence: Intervention 81.0 (24.9), Asthma education group 33.6 (27.4), Control 37.0 (32.3).	+44%	++	Parental conflict, functional severity index for asthma, spirometry	Intervention improved mean (SD) scores from 0.7 (0.7) to 0.5 (0.5); Education group from 1.4 (1.1) to 0.9 (0.8), and control from 1.2 (0.8) to 1.3 (1.0). Overall: Improved asthma control as measured by functional severity index
Elixhauser et al., 1990 [63]	Bipolar affective disorder	No	Simple EAM	Indirect	No	Self-report, Prescription refill, Levels	0 No difference in adherence from any measure was detected at any visit time point–the percentage of prescription refills was higher (82% vs 69%) in the monitored group between visit 1 and 2. These differences disappear after both groups received feedback (between visits 2 and 3) on adherence (74% intervention vs 81% control). Overall prescription refill rate (76% intervention vs 73% control). In the monitored group, adherence was 83.2% with monitoring alone, then with feedback (and monitoring), it declined to 76.0%. No SD data for the percentages of prescriptions refilled.	+3%	--	Symptom Questionnaire	Monitored group patients had higher adjusted scores for anxiety (P = 0.03), symptoms of depression (P = 0.02), and somatism (P = 0.03). No significant differences between groups’ hostility scale scores were detected. Overall: Worsened anxiety, depression and somatic complaints
Erickson et al., 2005 [45]	Hypertension	Not stated	Integrated	Direct	No	EM (intervention only), Adherence questionnaire (Morisky self report scores; both groups, used to compare adherence between groups)	0 The change in mean (SD) adherence between the 2 groups was not significant (0.13 (0.4) (control) vs 0.34 (0.6) (intervention); p = 0.20) though a significant increase in self-reported adherence *within* the intervention group compared to baseline was seen. This may be affected by the lower baseline adherence in the intervention group (4.62 (0.6) (baseline) 4.96 (0.1) (post-intervention); p = 0.02).	Not available	+	BP control	Mean (SD) change in Systolic BP: 3.4 (18.8) (control) vs –7.6(6.9) (intervention); p = 0.07 Mean (SD) change in Diastolic BP: 0.2(7.1) (control) vs –4.4(10.4) (intervention); p = 0.13 Overall: Greater BP lowering (non-significant favouring intervention group)
Forni Ogna et al., 2016 [50]	Percutaneous coronary intervention with stent	No	Simple EAM	Indirect	No	EM, self-report	++ Medication adherence was higher and less variable in the intervention group: median (min–max) Intervention 101 (94–102)% vs. 99 (83–101) % in the usual care group; P < 0.001. Median (min–max) correct adherence 99 (93–100) % (intervention) vs. 98 (80–100) % (usual care); P < 0.001.	+2%	0	Platelet reactivity index–vasodilator-stimulated phosphoprotein phosphorylation–platelet reactivity index (VASP-PRI)	Baseline mean VASP-PRI was 48.3 ± 18.8%. No significant difference between groups was observed (Mean (SD): 47.0 (15.8) control vs. 47.3 (19.2) intervention; p = 0.761. Overall: No effect on clinical
Foster et al., 2014 [11]	Asthma	Yes	Simple EAM	Both	Yes	EM	++ Digital group mean (SD) adherence = 71 (34.8)% vs. control = 46 (32.5)%; p = 0.0003	+27%	+	ACT, exacerbations	Mean (SD) ACT in intervention = 4.8 (4.53) vs control 3.6 (4.37); mean change overall 4.5 (4.9); p<0.0001). Severe exacerbations were experienced by 11% of the patients in the intervention group and 28% of the patients in control group (P = 0.013) Overall: Improved asthma control and improved exacerbations
Frick et al., 2001 [38]	HIV	No	Simple EAM	Direct	No	EM, Pill count, Self-report	++ Intervention group significantly more likely to have good adherence (defined as ≥95%) than those in the control group (82% vs 36%). Median rate of daily adherence was 100% in the intervention vs. 93% in the control (P<0.001); median rates of hourly adherence were 97% vs 87% in the intervention vs. control groups respectively (P<0.001).	+46%	Not measured	-	Not measured
Gregoriano et al. 2019 [35]	COPD, Asthma	No	Simple EAM	Both	No	EM	++ Mean (SD) percentage of days in target range of 80–100% adherence: 81.6 (14.2)% intervention vs 60.1 (30.3)% control; p<0.001 for puff inhalers; 89.6 (9.8)% intervention 80.2 (21.3)% control; p = 0.01 for dry powder inhalers	+22% (puff inhalers); 9% (dry powder inhalers)	+	Exacerbations	Longer average time to the next exacerbation was observed in the intervention compared to the control group (102 days [95% CI, 76 to 128] vs. 86 days [95% CI, 66 to 106], p = 0.19). Intervention had no effect on time to first exacerbation (HR 0.65, 95% CI 0.21 to 2.07, p = 0.24), but showed a trend toward a 39% decreased frequency of exacerbations (RR = 0.61, 95% CI 0.35 to 1.03, p = 0.07). No significant differences in QoL were found between the two groups. Overall: Decreased frequency of exacerbations; longer average time to next exacerbation (trend only; not significant)
Hardstaff et al., 2003 [57]	Renal transplant	No	Simple EAM	Indirect	No	EM	0 No difference in adherence was noted between the groups, either before or after feedback (26% improved in feedback group vs 20% in control; 26% improved post-feedback vs 8% in control).	Not available	Not measured	-	Not measured
Henriksson et al. 2016 [58]	Renal transplant	Not stated	Simple EAM	Direct	Yes	EM (only in intervention group) (adherence not assessed in control group)	0 Adherence 97.8% in the intervention group. No comparison data as medication adherence in the control group was not assessed (group did not use EAM).	Not available	+	Rejection rate	Non significant positive intervention effect: Biopsy-verified rejection was three times more common among controls (13 vs. 4 patients; p = 0.054). Overall: Lower rejection rates (trend)
Hermann et al., 2011 [68]	Glaucoma	Yes	Simple EAM	No feedback	No feedback	EM	0 No difference in mean (SD) adherence between intervention and control groups (70 (17)% intervention vs 77 (6)% control for twice daily dosing; 65 (14)% intervention vs 62 (9)% control for three times daily dosing; p = 0.24).	-7% (BD dosing), +3% (TDS)	Not measured	-	Not measured
Joost et al., 2014 [51]	Renal transplant	No	Simple EAM	Indirect	No	EM, Pill count, Self-report, Q	++ Mean adherence (91% (95% CI, 90.52–91.94) intervention vs 75% (95% CI, 74.57–76.09) control, p = 0.014)	+16%	0	Transplant function (eGFR), transplant rejections, SF36 (HRQOL), HADS-D for depression/ anxiety	Two patients had rejection in the control and one in the intervention group (p = 0.54). Transplant function improved in both groups and were similar at study end (Intervention 49 (14.3) mL/min vs control 46 (15.4) mL/min; p = 0.446. Overall: No effect on clinical
Kozuki et al., 2006 [59]	Psychotic disorder	Not stated	Simple EAM	Indirect	No	EM, Drug levels, Pill count	++ Adherence rates of the intervention group slightly increased compared to a decline in the control group (P = 0.026) (rates at 12 weeks were 87.9% vs 68.4% intervention vs control).	+20%	+	Positive and Negative Symptom Scale (PANSS)	PANSS positive scores: Change from 20.7 (2.6) to 20.0 (3.9)(intervention) vs. 21.2 (4.3) to 23.0 (3.5)(control). PANSS negative scores: Change from 18.2 (4.1) to 17.9 (2.9)(intervention) vs. 18.7 (3.3) to 19.7 (4.0) (control). Neither change statistically significant between the two groups. Overall: Improved PANSS (trend, not significant)
Matteson-Kome et al., 2014 [26]	Inflammatory bowel disease	Not stated	Simple EAM	Indirect	No	EM	0 MEMS change scores for the intervention group increased (MEMS difference mean = −0.07; SD = 0.03) and the MEMS change scores for the control group decreased slightly (MEMS difference mean = 0.01, SD = 0.06). Between the two groups, the change scores were not statistically significant (P = 0.14; CI: -0.19–0.045).	Not available	Not measured	-	Not measured
McKenney et al., 1992 [46]	Hypertension	Yes	Simple EAM	Direct	Yes	Pill count	++ Intervention group had a higher average adherence (95.1% vs 78%; p = 0.0002); less variation in adherence (62–111% vs 16–110%); lower % of non-adherent patients taking ≤80% of doses (8% vs 50%).	+17%	++	BP control	Intervention had greater mean (SD) decreases in systolic BP (-7.64 (17.24) mmHg, p = 0.006 vs -2.79 (13.82) mmHg, p = 0.13) and diastolic BP (-8.78 (8.93), p<0.0001 mmHg vs -0.24 (7.50) mmHg, p = 0.43). Overall: Greater reduction in BP
Mehta et al., 2019 [49]	Hypertension	Not stated	Simple EAM	Direct	No	EM, Self-report (intervention group only–adherence not measured in control group)	n/a Only medication adherence reported for intervention group (70.8%); not measured in control	n/a	0	BP	Intervention mean (SD) change in systolic BP: -4.3 (21.5) vs control -4.7 (23.4); p = 0.94 Intervention mean (SD) change in diastolic BP: +6.5 (15.2) vs control +4.0 (12.6); p = 0.44 Overall: No effect on clinical
Morton et al., 2017 [32]	Asthma	No	Simple EAM	Both	Yes	EM	++ Intervention group mean (SD) = 70 (22.8)% vs control = 49 (26)%; p<0.001	+21%	++	ACQ, Oral steroid use, hospital admissions, FEV%	Mean (SD) ACQ: Intervention 1.14 (1.37) vs control (0.95 (1.37). Overall: Decrease oral steroid use and hospital admissions, but no effect on ACQ or FEV%
Murray et al., 2007 [52]	Congestive Heart Failure	Not stated	Simple EAM	Indirect	No	EM, Prescription refill, Self-report	++, 0 on f/u Overall taking adherence was 67.9% and 78.8% in the usual care and intervention groups, respectively (difference, 10.9 percentage points [95% CI, 5.0 to 16.7 percentage points]); but these effects disappeared in the 3-month post-intervention phase in which taking adherence was 66.7% and 70.6%, respectively (difference, 3.9 percentage points [CI, -5.9 to 6.5 percentage points])	+11%	++	HF exacerbations requiring ED/hospital admission, costs, disease specific quality of life using the CHF questionnaire	Emergency department visits and hospital admissions were overall 19.4% less (for all causes of admission, mean (SD) 2.94 (4.69) intervention vs 3.65 (6.26) control group; risk ratio 0.82, 95% CI 0.70–0.95). QoL improved from BL to 6- and 12-months to a greater extent in the intervention group (0.28 vs 0.21 at 6m (P = 0.52); 0.39 vs 0.24 at 12m (P = 0.21)) Overall: Reduced ED and hospital visits, greater improvement in QOL and patient satisfaction
Nides et al., 1993 [65]	COPD	Yes	Simple EAM	Indirect	No	EM, Self-report, Canister Weights	++ Mean (SD) % adherence: intervention 88.8 (9.6)% vs control 68.8 (25.7)%; p<0.0001	+20%	Not measured	-	Not measured
Okeke et al., 2009 [71]	Glaucoma	No	Simple EAM	Direct	No	EM, Self-report	++ Mean (SD) intervention group adherence 73 (22) vs. control 51 (30)%.	+22%	0	Intraocular pressure	Intraocular pressure did not change significantly from baseline in either groups (p = 0.81). Overall: No effect on clinical
Onyirimba et al., 2003 [31]	Asthma	No	Simple EAM	Indirect	No	EM	++ Mean weekly inhaled steroid adherence over the first week was not significantly different (61 ± 9% vs 51 ±5% treatment and control respectively). By second week, adherence was 81 ± 7% (treatment group) vs 47 ± 7% (control) (P = 0.003). Adherence remained >70% in the treatment group for the entire trial but decreased in the control group to below 30%.	+34%	0	FEV1, Asthma QOL Questionnaire	FEV1 did not change significantly from baseline in either group (+0.04 ± 0.11L and +0.16 ± 11L for treatment and control groups, P = 0.44). QOL scores improved significantly from BL in both groups (1.13 ± 0.31 units and 0.76 ± 0.33 tx vs control groups; P<0.05) but there was no group difference in the degree of improvement (P = 0.43). Overall: No effect on clinical
Reddy et al., 2016 [60]	Coronary artery disease	Not stated	Simple EAM	Both	No	EM	++ Control group adherence rate: 0.67(0.60,0.75); Individual feedback (0.89 (0.81,0.97); Feedback with partner (0.86(0.80,0.92))	+20%	0	LDL levels	Change in LDL at 6 months: Control -9.14 (-19.47,1.18) vs. individual feedback group -5.01 (-15.49,5.48), p = 0.85 vs. feedback with partner -4.60 (-13.55, 4.35), p = 0.79. Overall: No effect on clinical
Rigsby et al., 2000 [40]	HIV	Not stated	Simple EAM	Indirect	No	EM, Drug levels	++, 0 on f/u There was a significant increase over time in adherence for the intervention group with cash reinforcement (P = 0.0005) but not the cue dose training group alone (P = 0.79). Adherence declined in the 8 weeks after discontinuation of training and reinforcement to near-baseline levels. The improvement in adherence in the cash-reinforced group was accompanied by a significant loss of these gains in the follow-up period.	Not available	0	Viral load	There was no differences in viral load change between each of the groups (+0.64 in the reinforcement group, -0.29 in the cue dose training group, +0.34 control). Overall: No effect on clinical
Rosen et al., 2004 [53]	Diabetes	No	Simple EAM	Both	Yes	EM, Self-report	++ Mean improvement in adherence of 15% in the intervention group to metformin (~60% increased to 80% but control group remained at ~60%; P = 0.017); improvements noted for other antihyperglycemic medications too but as only 17 patients were on a second medication, this was not significant (P = 0.50). No difference between group when self-report used.	+15%	0	HbA1c	There was little change in either group’s HbA1c from baseline and HbA1c did not differ between groups. Overall: No effect on clinical
Rosen et al., 2007 [39]	HIV	Not stated	Simple EAM	Indirect	No	EM, Self-report, Drug levels	++ Mean MEMS-measured adherence to the reinforced medication increased from 61% to 76% in the 16-week treatment phase and was higher than the supportive counselling group (59% to 44%) (P = 0.01). This difference wast lost in the 16 weeks of follow-up—no difference between the groups (P = 0.07). Those receiving the intervention were more likely to achieve 95% adherence in weeks 1–16 than the control (P = 0.02).	+32%	++	Viral load, CD4	Proportion of patients with viral load <400 HIV-RNA/mL: Intervention 13/18 (72%) vs control 10/21 (48%); p = 0.12 At follow up: intervention 9/14 (64%); control 12/17 (71%); p = 0.71 Overall: Reduced viral load, but improvements lost on follow-up
Ruppar, 2010 [47]	Hypertension	Not stated	Simple EAM	Both	Yes	EM	++, 0 on f/u Treatment group had better antihypertensive medication adherence than the control group (median adherence improved 15.4% with the treatment group whereas the control group had -5.6% in adherence, P = 0.003). Change during the intervention from 75.5 to 96.4% (int) vs 34.1 to 16.40% (control); at week 20 (after 12 weeks follow-up) the intervention adherence = 94.3% vs 40% in control, p = 0.206.	+80%*	++	BP control	The intervention group’s median systolic BP lowered to 130 mm Hg (interquartile range (IQR), 17 mm Hg) vs. control where the median SBP increased to to 152 mm Hg (IQR, 61 mm Hg); p = 0.008). Overall: Greater reduction in BP
Russell et al., 2011 [54]	Renal transplant	Not stated	Simple EAM	Indirect	No	EM	++ Mean medication adherence score for intervention group higher than control (P = 0.03) with a large intervention effect size (Cohen’s d = 1.4; r = 0.6).	Not available	Not measured	-	Not measured
Sabin et al., 2010 [41]	HIV	No	Simple EAM	Indirect	No	EM, Self-report	++ Mean (SD) adherence at month 12 mean adherence had increased to 96.4 (3.4)% in the intervention group but was unchanged in the control at 84.1 (21.4)% (P = 0.003).	+12%	+/-	Viral load, CD4	A higher proportion of subjects experienced an increase in CD4 (71.0%) than the control (48.4%)(P = 0.07). Mean CD4 count at month 12 rose by 90.0 (171.6) cells/microlitre in the intervention group but declined by 8.8 (152.6) cells/microlitre in the control (P = 0.02). Proportion of subjects with HIV RNA <400copies/mL at month 12 did not differ significantly: 27/31 (87.1%) intervention vs 31/33 (93.9%); p = 0.3518. Overall: Improved CD4 count but not viral load
Smith et al., 2003 [42]	HIV	No	Simple EAM	Indirect	No	EM	++ Individuals in the intervention group significantly more likely to take 80% of more of their doses each week than individuals in the control group (OR = 7.8, 95% CI 2.2–28.1). Average weekly adherence was higher in the intervention group at all time points. Average adherence increased over time and by 12 weeks average weekly adherence in the intervention group was 96% vs 37% in the control group.	+59%	+	Viral load, CD 4	41% (9/22) of individuals in the Intervention group had at least one viral load of 400 copies or less vs. 24% of individuals (5/21) in the control group; p = 0.27. Overall: Reduced viral load (Trend, not significant)
Sulaiman et al. 2018 [33]	Asthma	Not stated	Simple EAM	Indirect	No	EM	++ Mean (SD) adherence: 73 (24) % (intervention) vs 63 (26) % (control), p<0.01	+10%	0	Asthma control	Data on peak flow, ACT, quality of life and adherence combined to produce asthma control measure. Reported no difference between groups Overall: No effect on clinical
Sutton et al., 2014 [61]	Diabetes	No	Simple EAM	No feedback	No feedback	EM (intervention only), Adherence questionnaire (both groups; used to compare adherence between groups)	0 Self-reported mean (SD) adherence using adherence questionnaire: intervention 24.2 (1.1) vs control 23.8 (1.9); p = 0.11.	Not available	+	HbA1c	Intervention mean (SD): 8.22 (1.30)% vs control: 8.39 (1.16); p = 0.25. Overall: Improved HbA1c (trend, not significant)
Tashkin et al., 1991 [66]	COPD	Yes	Simple EAM	Indirect	No	EM, Self-report, Canister Weights	++ In the control group 87% self-reported inhaler use at least twice a day, but only 52% had used it two or more times daily as per the chronology EM. In the feedback group, 89% self-reported adherence, but only 78% had used the inhaler two or more times daily from the chronology EM. The proportion of feedback participant with satisfactory compliance was significantly greater than the proportion of compliant uninformed participants (P<0.0001).	+26%	Not measured	-	Not measured
van Onzenoort et al., 2012 [48]	Hypertension	No	Simple EAM	No feedback	No feedback	EM, Pill count	0 Based on pill counts—median adherence did not differ between the two groups (96.1% (88.8–98.4 interquartile range) (intervention) vs 94.2% (control); P = 0.97).	+2%	0	BP control	Mean (SD) difference in systolic BP: intervention 23 (23) mmHg vs 22 (19)mmHg, p = 0.42 Mean (SD) difference in diastolic BP: intervention 13(13) mmHg vs 12 (11) mmHg, p = 0.62 Overall: No effect on clinical
Vasbinder et al. 2017 [34]	Asthma	No	Simple EAM	Direct	Yes	EM	++ Adherence in the intervention group was 69.3% (95% CI 65.5–73.4%) and 57.3% (95% CI 52.8–61.7%) in the control group. The overall difference was statistically significant: 12.0% (95% CI 6.7–17.7%)	+12%	0	Asthma control, QOL, exacerbations	No significant change in c-ACT (-1.07 (95% confidence interval -3.51–0.56),p = 0.203; quality of life as per PAQLQ (-.-6 (= 0.41–0.15,p = 0.659); and exacerbations per year (= 0.14 (-0.61–0.25,p = 0.432) Overall: No effect on clinical
Velligan et al., 2013 [70]	Schizophrenia	Not stated	Integrated	Both	No	EM, Pill count	++ Mean (SD) adherence 91 (17.46)% for intervention vs. 72 (17.26)% for the control group.	+19%	0	Brief Psychiatric Rating Scale (BPRS), ED/ hosp use, Social and Occupational Functioning Scale (SOFAS) for global function	For SOFAS scores and BPRS, results of mixed-effects regression models yielded no significant main effects or interactions (all P values > .09). For ED use, 13 out of 47 patients (27.7%) in the intervention group, and 16 out of 47 (34%) in control had contact with hospital or emergency psychiatric services; p = 0.77. Overall: No effect on clinical
Wilson et al., 2010 [43]	HIV	Not stated	Simple EAM	Indirect	No	EM, Self-report	0 There was a trend toward an intervention effect; adherence was 2.0% (95% CI -1.95 to 5.9) higher in the intervention group but not significant (P = 0.32).	+2%	0	Viral load	No significant differences in viral load–data not reported Overall: No effect on clinical
Wu et al., 2006 [44]	HIV	Not stated	Integrated	Direct	No	EM	++ Adherence was improved with 77% vs 57% (see Andrade et al.)	+20%	-	QOL scores, instrumental activities of daily living (IADLs), Centers for Epidemiologic Studies Depression Scale for depression, Medical Outcomes Study HIV Health Survey (MOS-HIV) for QOL	MOS-HIV scale–quality of life scores: -10.61 (intervention) vs. 8.05 (control); p = 0.06 IALDs: 1.48 (intervention) vs. -1.79 (control); p = 0.02 (higher scores, worse health) Depression scale: -1.54 (intervention) vs. -7.45 (control); p = 0.03 (higher scores, worse health) Overall: Worsened QOL (trend, not significant)
Yeh et al., 2017 [55]	Multiple sclerosis	Not stated	Simple EAM	Indirect	No	EM, Prescription refill, Self-report	0 Intervention had worse adherence than control using MEMS at 3 months Cohen’s d (-0.34) and 6 months (-0.7).	n/a	Not measured	-	Not measured

^#^++ (significant improvement), + (trend in improvements), +/- (improvement in some parameters but not others), 0 (no effect),—(trend towards worsening), — (significant worsening)

ACQ = Asthma Control Questionnaire; ACT = Asthma Control Test; BP = blood pressure; CI = confidence interval; COPD = chronic obstructive pulmonary disease; EAM = electronic adherence monitoring; EM = electronic monitoring; HF = Heart Failure; HIV = Human Immunodeficiency virus; QoL = Quality of Life

Due to the wide heterogeneity of the types of measures used to assess clinical outcomes, meta-analyses could only be conducted by disease group, where there were three or more studies reporting on clinical effect in a similar way. Across the 38 studies that reported on clinical outcomes, meta-analyses could be conducted using the following outcome measures–viral load for HIV, blood pressure for hypertension; and asthma control measures for asthma. For studies in HIV, 5 [36, 37, 72–74] of the 9 HIV studies reported on proportion of patients with undetectable viral load as an outcome;. In hypertension, 4 [49, 75–77] of the 7 studies reported on mean change in blood pressure (systolic and diastolic); and in asthma, 5 of the 9 studies reported on change in asthma control [28, 32, 34, 78, 79], in a way that could be included in the meta-analysis. Table 4 shows the evidence profile for these three clinical outcomes, and for adherence.

**Table 4:** Evidence profile for adherence and clinical outcomes in HIV, hypertension and asthma.

Certainty assessment							№ of patients		Effect		Certainty	Importance
№ of studies	Study design	Risk of bias	Inconsistency	Indirectness	Imprecision	Other considerations	EMM interventions	usual care	Relative (95% CI)	Absolute (95% CI)		
**Adherence**												
27	randomised trials	serious^a^	serious^b^	not serious	not serious^c^	strong association	1267	1317	-	SMD 0.93 higher (0.69 higher to 1.17 higher)	⨁⨁⨁◯ Moderate	IMPORTANT
**Proportion of patients with undetectable HIV viral load**												
5	randomised trials	not serious^d^	not serious^e^	not serious	very serious^f^	none	117/164 (71.3%)	107/167 (64.1%)	not estimable		⨁⨁◯◯ Low	CRITICAL
**Change in blood pressure from baseline—Systolic BP**												
4	randomised trials	not serious^g^	serious^h^	not serious	serious^i^	none	340	326	-	MD **2.2 lower** (6.99 lower to 2.59 higher)	⨁⨁◯◯ Low	CRITICAL
**Change in blood pressure from baseline—Diastolic BP**												
4	randomised trials	not serious^g^	serious^j^	not serious	serious^i^	none	340	326	-	MD **2.44 lower** (7.8 lower to 2.92 higher)	⨁⨁◯◯ Low	CRITICAL
**Change in asthma control from baseline**												
5	randomised trials	serious^k^	not serious	serious	serious^l^	all plausible residual confounding would reduce the demonstrated effect	309	335	-	SMD 0.09 higher (0.07 lower to 0.24 higher)	⨁⨁◯◯ Low	CRITICAL

CI: confidence interval; SMD: standardised mean difference

Explanations

a. Allocation concealment was unclear in most studies, with most studies at high risk of performance bias due to the inability to blind participants to the EMM intervention. This may have affected adherence behaviour in participants over and above the EMM intervention effect

b. Imprecision—heterogeneity is high with I-squared = 86%

c. The boundaries of the CI are on the same side of their decision-making threshold and generally consistent across studies

d. Most studies included that assess this clinical outcome have unclear selection bias and are at high risk of performance bias due to the inability to blind participants to the EMM intervention. However, the clinical outcome (viral load) in this case is objective and therefore at low risk of detection bias.

e. Studies generally consistent with an I-square statistic of 40% suggesting studies generally homogenous

f. Studies generally small and events are few; risk difference is low with the confidence interval crossing zero

g. Included studies assessing this clinical outcome have unclear risk of selection bias, but generally low risk of bias in the other domains. Of note, the outcome blood pressure is unlikely to be affected by performance or detection bias

h. Although I-square is 41%, there is some inconsistency between studies

i. Relatively wide confidence interval crossing the no effect line, sample size less than 400

j. High heterogeneity in studies for diastolic BP outcomes, with I-square of 86%

k. Studies are at unclear risk of selection bias and high risk of performance and detection bias due to the nature of the EMM intervention. The outcome of interest is self reported asthma control; as such has potential to be affected by knowledge of intervention group

l. Wide confidence interval that crosses no effect line with relatively few number of studies

There were 3 studies that reported outcomes in transplant patients, however only 2 reported numbers of patients with rejection [51, 58] while the other reported on 5-year event-free survival rates [64]. The remaining 11 studies reporting on outcomes were in a range of health conditions: heart failure (2/10); diabetes (2/10); glaucoma; bipolar disorder; percutaneous coronary intervention; psychosis; COPD; coronary artery disease; and schizophrenia, all reporting on outcomes using different measures (e.g. symptom scores or hospitalisation rates) and therefore could not be synthesised via meta-analysis.

**Fig 3:**
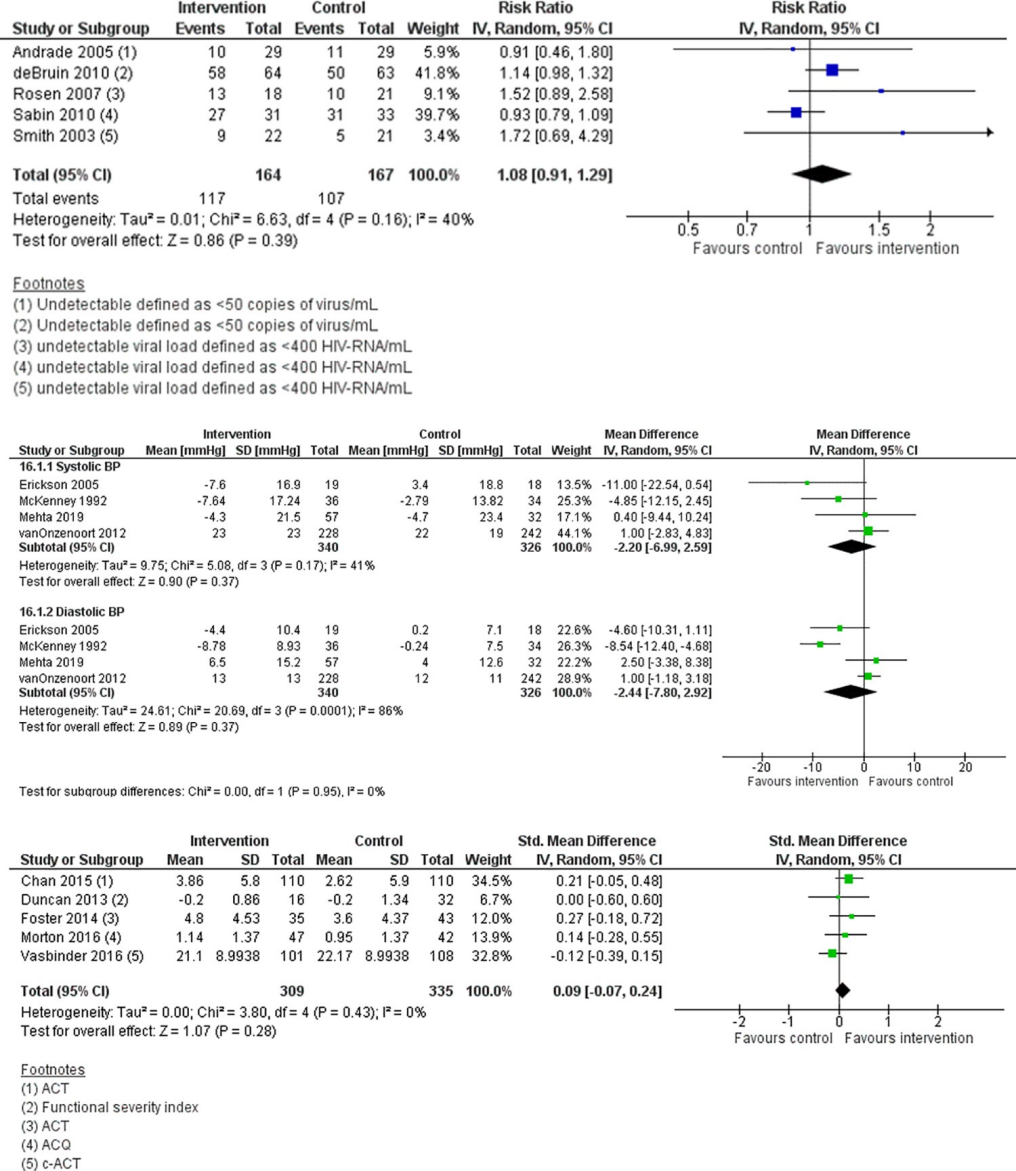
Forest plot of effect of the electronic adherence monitoring intervention compared to control on clinical outcomes by a) viral load for Human Immunodeficiency Virus; b) blood pressure for hypertension; c) asthma control for asthma.

Fig 3 shows the effect of EAM on clinical outcomes in HIV, hypertension and asthma. For all outcomes, the analysis crossed the boundary of now effect, but showed a non-significant effect favouring the EAM group. For HIV, those receiving the intervention had a 1.08 (95% CI 0.91–1.29, p = 0.39) chance of having an undetectable viral load. In hypertension, the EAM group achieved a lower systolic and diastolic blood pressure by 2mmHg, though the 95% CI was wide. For asthma, a SMD of 0.09 (95% CI, -0.07–0.24, p = 0.43) was seen, indicating a small but positive improvement in asthma control favouring the intervention, though this was not significant. Table 3 describes narratively the outcomes for other health conditions, which report a range of effect from significant improvements in outcome to no effect. There were 15 studies that reported no effect on outcomes; these had improvements in adherence from 2% [43, 48] to 34% [31], and had a lack of blinding or real-time feedback, with most (11/15 studies) not feeding back adherence in real-time to the participant.

Not all studies reporting clinical benefit had improvements in adherence. Four studies [45, 58, 61, 64] had no significant effect on adherence but reported clinical benefit. Three [36, 41, 69] had improvements in some but not all clinical parameters (e.g. reduced viral load, but no effect on CD4 count [36]), and two [44, 63] had a negative effect on clinical outcomes but a positive [44] or no effect [63] on adherence. In a study of patients with bipolar affective disorder, the EAM intervention group reported higher rates of anxiety, depression and somatism than the controls [63]. Wu et al. reported worsened quality of life in patients with HIV who received the EAM intervention [44] than those who did not, but no differences in disease control.

### Assessment of risk of bias

Most studies had at least one domain rated as having a high risk of bias (n = 40, 85%, S1 and S2 Files). There was a high performance bias in most studies (n = 35, 74%). This is expected as blinding of patients and health professionals is difficult due to the nature of the EAM intervention. Most studies (n = 38, 81%) had low detection bias as adherence and clinical outcome measures were objectively measured. Whist the risk of selective reporting bias was low within each study, overall, there was moderate selective reporting bias as 20 of the 47 studies did not report data in a way that could be meta-analysed.

### Assessment of publication bias across studies

The funnel plot (S3 File) indicated acceptable plot symmetry for both adherence and clinical outcomes data, suggesting limited publication bias, though the large amount of study heterogeneity needs to be considered.

### Quality of evidence

Table 4 shows the GRADE ratings for adherence and clinical outcomes for the 3 conditions where this could be meta-analysed.

Risk of bias for adherence (S1 and S2 Files) was rated as serious for the adherence outcome due to the high number of studies with unclear allocation concealment and the risk of performance bias, as a results of the inability to blind the intervention to the participants and outcome assessors in most studies, which could affect outcome reporting. For clinical outcomes however, the nature of the outcomes (viral load and blood pressure) are objective measures that are unlikely affected by knowledge of group allocation; as such evidence was not downgraded for HIV and hypertension but was so for asthma. Inconsistency was downgraded and rated as serious for adherence and blood pressure, given the high heterogeneity of the studies.

Indirectness, imprecision and publication bias were not downgraded for adherence, but evidence was downgraded for risk of bias and inconsistency. Quality was upgraded given the strong association and large effect size. This gave an overall rating of moderate certainty in the effect estimate for adherence. For clinical outcomes, overall certainty in the evidence was deemed low, mainly as a result of inconsistency and imprecision as a result of the small sample sizes per condition.

### Patient acceptability perceptions of the EAM intervention

Fourteen studies evaluated patient acceptability of the EAM intervention (see S4 File). Nine assessed usability of the device [11, 30, 37, 38, 44, 49, 67, 69, 70] though one study did not report the results [67]; four focused on the acceptability of the adherence feedback and interaction with health providers [47, 53, 54, 56]; and one study focused on both the device and the health provider interaction [62].

#### Patient perceptions of EAM

Of the nine studies that reported patient perceptions of the device, perceptions were negative in nearly half [37, 44, 69, 70]. In the Velligan et al. study, participants preferred getting medication support from staff rather than from EAM [70], and had negative feedback, primarily about the device’s reminder beeps. Similarly, Wu et al. reported that the EAM device was too large and too loud, leading to unwelcome questions and possible revelation of the patient’s HIV status [44]. In contrast, the EAM device used by Frick et al., which included a button to silence the alarm, received positive feedback, with 99% stating they would use the vial again and 97% finding the alarm helpful [38]. Other studies reported mixed results, with some participants enjoying EAM as they felt it helped them stay on schedule with their doses, whilst others “hated the device” and felt their lives were regulated by the EAM device, and found EAM to be a nuisance [69], unnecessary and unattractive [37, 80].

#### Patient perceptions of the adherence intervention

Patient perceptions of the adherence intervention were more positive than for the device [37]. Patients and health providers found the adherence review and discussions the most beneficial parts of the intervention and “looked forward” to receiving their adherence data [47, 53, 56, 62]. The adherence feedback did not make patients feel uncomfortable [53] and was not perceived to be intrusive [62], or a burden [54].

#### Other findings

Although most studies did not aim to evaluate patient perceptions, four studies attributed recruitment issues, patient drop-outs and non-participation to issues with device acceptability by patients (S4 File) [14, 37, 45, 51]. Training was also identified as a factor to consider for intervention acceptability [41].

## Discussion

This is the first systematic review and meta-analysis of EAM and the effect on medication adherence and clinical outcomes, across all chronic conditions. To our knowledge, this systematic review and meta-analysis is the largest in this field, comprising 47 RCTs in the systematic review and 27 studies in the meta-analysis of adherence. Whilst there have been systematic reviews across conditions, these have been for multiple types of interventions rather than specifically examining the effect of EAM. Patients receiving an EAM intervention had significantly better adherence compared to those who did not, with a large magnitude of effect (SMD = 0.93). SMD measures effect when studies report efficacy as a continuous measurement, with zero meaning the intervention and control groups have equivalent effects, with SMD increasing as the difference between the intervention and control group increases. An SMD over 0.8 is considered a large effect [81]. Putting this into perspective, when SMD = 0, the probability that the intervention outperforms control is 0.5 (no better than chance); and when SMD = 1, the probability increases to 0.76. In this review, SMD = 0.93, meaning for individuals who receive EAM, there is a approximately a 0.7 probability that their adherence will improve than if they didn’t receive the EAM. As our review found however, this may not consistently translate to clinical benefits, as this appears to vary depending on the population and chronic condition. This highlights the potential of EAM to improve medication adherence in patients with chronic conditions. Similar effect sizes were seen in studies measuring only taking adherence, and in studies using objective adherence measures. We found that the effect of EAM appeared particularly large for asthma and HIV, similar to findings from other reviews, though the number of studies per condition are small, which limits our confidence in the findings. Lee et al. reported that in children with asthma, those receiving an EAM intervention were 1.5 times more likely to adhere than those in the control group [18]. Similarly Christensen et al. noted high adherence rates reported across included studies for HIV populations receiving EAM [16], though the authors did not conduct a meta-analysis. Our findings are similar to previous reviews of the effect of reminders or adherence feedback on adherence, but the magnitude of effect in prior reviews was smaller and non-significant [82] or not quantified if findings in studies that only reported results narratively [7, 9, 10, 12, 13]. Previous reviews may have only been limited to one health condition [17, 18], or evaluated only one aspect of EAM (e.g. reminders, adherence feedback, or the packaging), which may explain our larger magnitude of effect. We found improvements in clinical outcomes in HIV, hypertension and in asthma, but none of these reached statistical significance due to the small number of studies that were able to be included.

There are several implications for physicians, researchers, and payers. First, our review found that intervention complexity was important for intervention effectiveness. Studies using an EAM device by itself, without reminders or health provider input, did not improve adherence. This aligns with previous literature showing that complex interventions–i.e. those involving more than one intervention element–are more effective [83]. A systematic review of electronic packaging interventions on adherence, including both RCTs and non-RCTs, found that complex interventions with EAM were the most effective for improving adherence [84]. Second, the delivery format of the EAM intervention did not appear to greatly influence the magnitude of effect. Intervention effectiveness was not influenced by how adherence feedback was provided or the timing of the feedback, nor by the age of the participants, with EAM being effective in both children and adults.

Third, although there were significant improvements in adherence, few RCTs reported corresponding benefits in clinical outcomes. Those that did show clinical improvements reported a greater magnitude of increase in adherence. It is possible that a minimum threshold of percentage adherence change is needed before any clinical change can be achieved, however the threshold is unknown for most conditions and depends on the medication pharmacology [85]. Whilst there are many studies demonstrating the association between adherence and clinical outcomes [86, 87], it is not known whether the relationship is a linear, exponential or logarithmic one, and the relationship is likely to be affected by the disease, medication and patient [85]. We found that, on average, only half of those interventions that improve adherence translate to corresponding improvements in clinical outcomes.

There are several limitations to consider. The impact on adherence was pooled from 27 studies; over 40% of the studies (20/47) did not report adherence data in way that could be meta-analysed. The impact on clinical outcomes is also less clear. Several studies did not measure clinical outcome data or where data were measured, the outcomes were not relevant to the condition. For example, Elixhauser et al. used a general psychological symptom questionnaire [88] to assess the effect of an intervention to improve lithium adherence, rather than a validated mania scale, which would have better reflect lithium adherence and response [63]. Even in conditions where disease control can be easily measured and validated disease control questionnaires exist, such as in asthma, heart failure, or diabetes, there was large heterogeneity in the measures used and outcomes studied that precluded inclusion in a meta-analysis [11, 14, 27–31, 45–48, 52, 53, 61, 69]. This lack of standardisation in outcome measures across the same disease state makes inter-study analyses and comparisons difficult. In this review, we performed a meta-analysis to synthesise clinical outcomes but only where meta-analysis was meaningful. As outcome measures were highly varied, we opted only group together measures for the same condition where these made clinical sense. The limitation of this approach is the small number of studies that were able to be included, which reduced our confidence in the findings. Additionally, for the studies that could not be included in the meta-analysis, this could only be described narratively and whether studies reported statistically significant benefits. This has limitations as it does not provide information about overall effect size. Questions also remain about the sustainability of the intervention effects–only 21 of the 38 studies that assessed clinical outcomes were of 6 months duration or longer. Whether these benefits are maintained in the long-term for chronic conditions is not known as initial intervention effects may wane over time. Whilst most studies assessed adherence well, by triangulating data from more than one objective adherence measure to evaluate adherence, the measurement of clinical outcomes is less consistent. Future research should use validated markers of disease control that can be used in different research studies and clinical settings. As adherence is only a mediator of therapeutic outcomes, adherence studies should ideally always include a measure of clinical outcomes as an endpoint, as achieving adherence is meaningless if patients are not getting any clinical benefits. This is seen in the studies that reported clinical benefits, but no effect on adherence. Of note, a limitation for our review is how quality of evidence was assessed. We used the Cochrane Risk of Bias 1.0 tool for evidence certainty grading; however, we note that random sequence generation and allocation concealment were often rated as ‘unclear risk of bias’ due to absence of sufficient detail in reported studies. If the Cochrane Risk of Bias 2.0 tool were used, this may potentially downgrade the evidence certainty to ‘very serious’ risk of bias.

Our results emphasise the need to consider the patient in healthcare interventions. Less than a third of the studies reported on patient acceptability, yet findings show that patient perceptions of the devices were often negative. In contrast, patient perceptions about the adherence interventions were positive, particularly about the provision of adherence feedback and the opportunity to interact with a health provider. Issues relating to the loudness of the reminder and device size are common themes that future interventions involving EAM should consider. Devices where patients are able to silence reminders and / or personalise the reminder setting may be more acceptable [80]. Training resources need to be considered, particularly as technologies change and more EAM devices become available. Our findings highlight the importance of including feasibility and patient acceptability measures in future research.

By combining data from RCTs, our systematic review and meta-analysis found that EAM can have a significant effect on medication adherence in chronic conditions. How these adherence improvements translate into clinical benefit is less clear. A quarter of the studies reporting adherence improvements had corresponding clinical benefits. The lack of standardised outcome measures that reliably and accurately reflect disease control prevents us from definitively answering how EAM affects clinical outcomes. Future research should measure clinical outcomes using standardised and validated tools; be of adequate study duration to assess the sustainability of improvements in adherence and disease control in the medium- to long-term; and importantly, evaluate the patient acceptability of the EAM intervention.

## Supporting information

S1 Checklist (DOC)

S1 Appendix Search strategy. (DOCX)

S1 File Summary risk of bias graph (n = 27) for adherence outcome using Cochrane Collaboration’s tool for assessing risk of bias for randomised controlled trials. Studies are categorised as ‘Low risk’ of bias (green), ‘High risk’ of bias (red) or ‘Unclear risk’ of bias (yellow).
(DOCX)

S2 File Cochrane collaboration’s tool for assessing risk of bias for randomised controlled trials for adherence outcome. Studies are categorised as ‘Low risk’ of bias (+), ‘High risk’ of bias (-) or ‘Unclear risk’ of bias (?).
(DOCX)

S3 File (DOCX)

S4 File Summary of patient acceptability data on interventions. (DOCX)

S1 Data (XLSX)
