# Supplementary material for: Effect of electronic adherence monitoring on adherence and outcomes in chronic conditions: A systematic review and meta-analysis
Source: PLoS One. 2022 Mar 21;17(3):e0265715. doi: 10.1371/journal.pone.0265715 (PMC8936478; doi:10.1371/journal.pone.0265715)
Supplement: S1 Appendix — (DOCX) [file pone.0265715.s002.docx]

## **S1 Appendix. Search strategy.**

Previously published reviews in a similar topic area were used to guide the search strategy and the formation of search terms.

The following Boolean search was conducted for OVIDSP databases: (electronic monitoring.mp. OR electronics.sh OR remote monitoring.mp. OR monitoring device.mp. OR reminder device.mp) AND (medication adherence OR patient compliance).sh. OR patient compliance.mp. OR medication adherence.mp. OR adhere*.mp. OR complian*.mp. OR non-adhere*.mp. OR nonadhere*.mp. OR non-complian*.mp. OR noncomplian*.mp.) AND (intervention study.mp. OR intervention studies.sh OR randomized controlled trial.sh OR randomized controlled trial.mp. OR randomised controlled trial.mp. OR controlled clinical trial.sh OR controlled clinical trial.mp.).

A modified version of this search was used for CINAHL Plus: (monitoring device.mp. OR reminder device.mp OR electronic monitoring.mp. OR electronics.sh OR remote monitoring.mp.) AND (medication compliance OR patient compliance).sh. OR patient compliance.mp. OR medication adherence.mp. OR medication compliance.mp. OR adherence.mp OR compliance.mp OR non-adherence.mp OR nonadherence.mp OR non-compliance.mp OR noncompliance.mp OR Noncompliance (NANDA).sh OR Noncompliance of Therapeutic Regimen (Saba CCC) OR Noncompliance of Medication Regimen (Saba CCC) OR Noncompliance (Saba CCC) AND (intervention study.mp. OR experimental studies.sh OR randomized controlled trials.sh OR randomized controlled trial.mp. OR randomised controlled trial.mp. OR clinical trials.sh OR controlled clinical trial.mp.).
