# Supplementary material for: Effect of electronic adherence monitoring on adherence and outcomes in chronic conditions: A systematic review and meta-analysis
Source: PLoS One. 2022 Mar 21;17(3):e0265715. doi: 10.1371/journal.pone.0265715 (PMC8936478; doi:10.1371/journal.pone.0265715)

## S1 File. Summary Risk of Bias Graph (n=27) for adherence outcome using Cochrane Collaboration’s tool for assessing risk of bias for randomised controlled trials. Studies are categorised as ‘Low risk’ of bias (green), ‘High risk’ of bias (red) or ‘Unclear risk’ of bias (yellow).


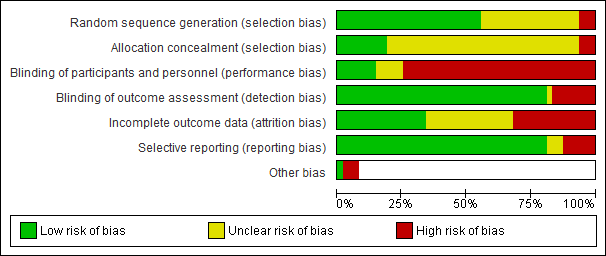

Supplement: S1 File — Studies are categorised as ‘Low risk’ of bias (green), ‘High risk’ of bias (red) or ‘Unclear risk’ of bias (yellow). (DOCX) [file pone.0265715.s003.docx]
