# Supplementary material for: Effect of electronic adherence monitoring on adherence and outcomes in chronic conditions: A systematic review and meta-analysis
Source: PLoS One. 2022 Mar 21;17(3):e0265715. doi: 10.1371/journal.pone.0265715 (PMC8936478; doi:10.1371/journal.pone.0265715)

## S2 File. Cochrane Collaboration’s tool for assessing risk of bias for randomised controlled trials for adherence outcome. Studies are categorised as ‘Low risk’ of bias (+), ‘High risk’ of bias (-) or ‘Unclear risk’ of bias (?).


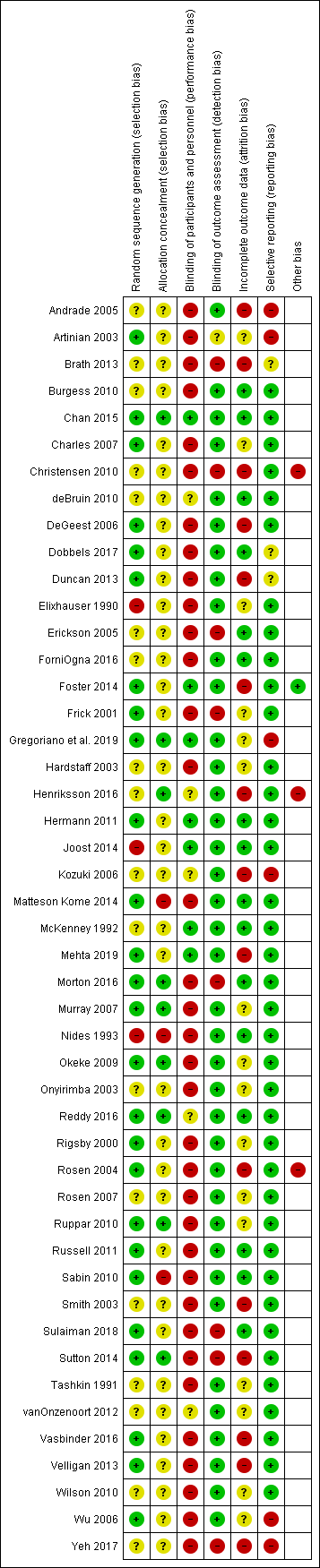

Supplement: S2 File — Studies are categorised as ‘Low risk’ of bias (+), ‘High risk’ of bias (-) or ‘Unclear risk’ of bias (?). (DOCX) [file pone.0265715.s004.docx]
