# Supplementary material for: Effect of electronic adherence monitoring on adherence and outcomes in chronic conditions: A systematic review and meta-analysis
Source: PLoS One. 2022 Mar 21;17(3):e0265715. doi: 10.1371/journal.pone.0265715 (PMC8936478; doi:10.1371/journal.pone.0265715)

## S3 File.

## Funnel Plot for all included studies for adherence (n= 27)


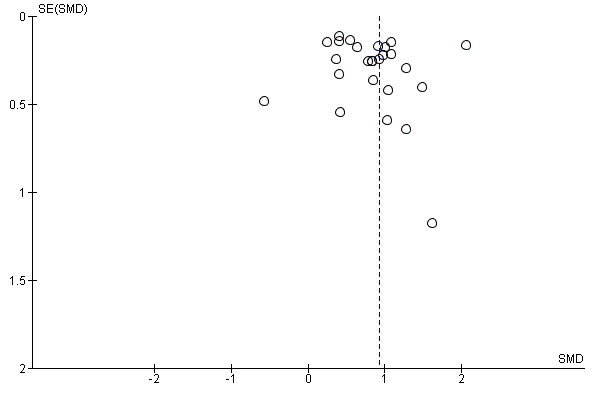


1. **Funnel plots for included studies in HIV, hypertension, and asthma**


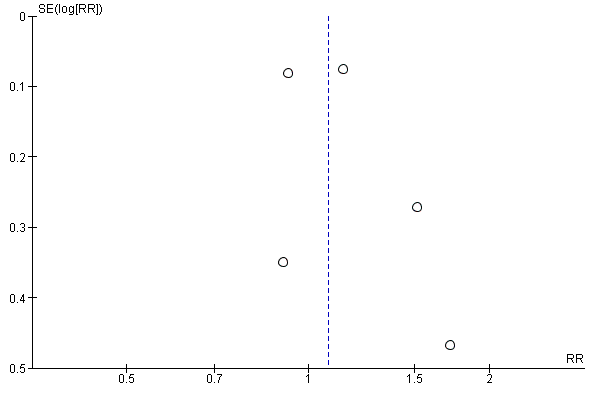


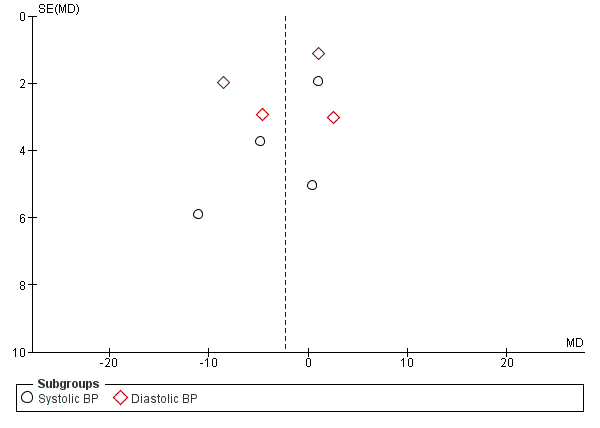


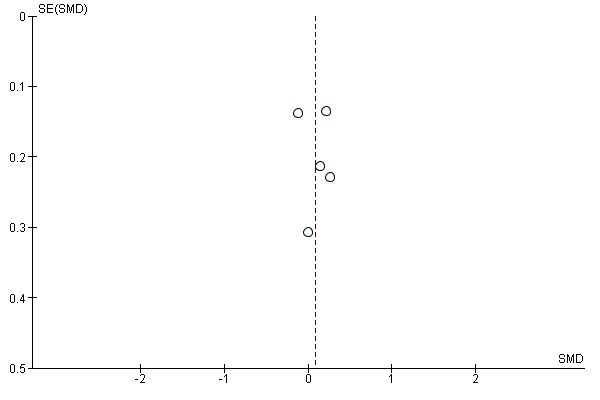

Supplement: S3 File — (DOCX) [file pone.0265715.s005.docx]
