# Supplementary material for: Effect of electronic adherence monitoring on adherence and outcomes in chronic conditions: A systematic review and meta-analysis
Source: PLoS One. 2022 Mar 21;17(3):e0265715. doi: 10.1371/journal.pone.0265715 (PMC8936478; doi:10.1371/journal.pone.0265715)
Supplement: S4 File — (DOCX) [file pone.0265715.s006.docx]

## S4 File. Summary of patient acceptability data on interventions.

| **Author** | **Patient acceptability data** | |
| --- | --- | --- |
|  | **Device-specific feedback** | **Intervention feedback** |
| Andrade et al., 2005 | Not assessed | |
| Artinian et al., 2003 | Patients loved it (56%) - found they enjoyed the reminders and were better able to stay on schedule with their medication doses; hated it (44%) as felt their life was regulated by the monitor and found it was a nuisance; some had technical difficulties (44%) with difficulties getting out the pills from the device or reading the screen; some felt it changed their lifestyle (67%) as it helped them establish a better routine (n=9). | N/a |
| Brath et al., 2013 | Patients were satisfied with the ease, speed and reliability of use, reporting that the EAM affixed to the medication blister did not disturb them. | Overall, the system was beneficial for the physician knowing if and when they are taking their medication. |
| Burgess et al., 2010 | Not assessed | |
| Chan et al., 2015 | Not assessed | |
| Charles et al., 2007 | Not assessed | |
| Christensen et al., 2010 | Not assessed, though a high percentage (50.3%) of patients chose not to use the device and were not included. | N/a |
| de Bruin et al., 2010 | Some patients refused to be in the study as the MEMS was too big (38/276); were afraid MEMS might disturb routine (17/276). After consideration, more patients felt the MEMS was too big and impractical to use (7/155) so refused to continue. At study end, another 6/133 patients dropped out due to the MEMS being too big or impractical to use. Evaluation by patients (n=50) and HIV nurses (n=7) about user-friendliness of MEMS-cap was scored 6.7 ± 2.8. Patients suggested they wanted the MEMS design to have multiple compartments for weekly management and a smaller, flat, pocket-sized pill box for day doses. | Usefulness of MEMS reports and intervention sessions with nurses was scored 8.2 ± 1.5. |
| De Geest et al., 2006 | N/a | Patients in the intervention group felt the EAM printouts were the most helpful part of the intervention to allow problem detection, feedback provision and goal setting. |
| Dobbels et al., 2017 | Not assessed | |
| Duncan et al., 2013 | Parental feedback on difficulty in using the MDILog-II (5.79 ± 0.97), usefulness of MDILog-II (5.93 ± 0.73) assessed on a 7-point Likert-type scale (1 - very negative; 7 - very positive). Youth reported a usefulness score of 5.50 ± 1.91. | N/a |
| Elixhauser et al., 1990 | Not assessed | |
| Erickson et al., 2005 | Not assessed but two intervention group patients dropped out in first few days of study due to lack of interest in using the device. | N/a |
| Forni Ogna et al., 2016 | Not assessed | |
| Foster et al., 2014 | Evaluated via qualitative interviews of a subsample of participants[80]. Mean satisfaction scores with the EAM was 82 (range 32-100). Three key themes were found relating to feasibility and acceptability of use of the device; utility and behavioural impact of reminders, and sustainability. | Participants felt the intervention led to a shift in adherence attitudes as the reminder helped them link adherence and asthma control. Use of the reminders was influenced by the clinician knowing the participant’s medication use. Some participants felt they wanted ongoing support after intervention had completed at 6 months. |
| Frick et al., 2001 | Vial acceptability was high - 99% stated they would use the vial again and felt it was easy to use. Majority in the alarmed group (97%) felt the alarm helped them to take their medication every day as directed. | N/a |
| Gregoriano et al. 2019 | Not assessed | |
| Hardstaff et al., 2003 | Not assessed | |
| Henriksson et al. 2016 | Six of the 40 participants in the intervention group withdrew from the study prematurely, 3 of them due to “a feeling of being monitored.” A few days after the start of the study, 1 participant experienced the EAM to be extremely stressful/worrisome, which resulted in the withdrawal from the study. None of the participants in the control group withdrew from the study prematurely. | N/a |
| Hermann et al., 2011 | Not assessed | |
| Joost et al., 2014 | 17/129 initial patients informed about the project did not want to participate due to use of MEMS; another 3/74 further patients withdrew after the study start due to denial of use of MEMS. | N/a |
| Kozuki et al., 2006 | Not assessed | |
| Matteson-Kome et al., 2014 | Not assessed | |
| McKenney et al., 1992 | Not assessed | |
| Mehta et al., 2019 | N/a | Participants in the pill bottle and text messaging arms were also asked “Please rate how likely you would be to recommend the Way2Text Program to others who are prescribe blood pressure medication on a scale from 0-10? (0 = Not At All Likely, 10 = Extremely Likely).”Among the 98 out of 117 participants in the pill bottle and text messaging arms who completed the follow up survey, there was a mean likelihood to recommend score of 9.0 out of 10 (SD 2.0), with a median of 10. |
| Morton et al., 2017 | Overall higher rates of broken, damaged and lost devices in the intervention group: 50% of devices were broken by the child in the intervention group vs 19% in the control; 37% were damaged beyond repair (intervention group) vs 5% in the control; 22% forgot their devices in the intervention group vs 43% in the control; and 11% lost the device completely in the intervention group vs 5% in the control. | N/a |
| Murray et al., 2007 | Not assessed | |
| Nides et al., 1993 | Not assessed | |
| Okeke et al., 2009 | Satisfaction with the devices was measured at 3- and 6- months but not reported. | N/a |
| Onyirimba et al., 2003 | Not assessed | |
| Reddy et al., 2016 | Not assessed | |
| Rigsby et al., 2000 | Not assessed | |
| Rosen et al., 2004 | A significantly higher proportion of patients in the intervention group knew that the MEMS caps recorded the date/ time (87.5% vs 11.8%, P<0.001). | Providers and patients also rated the process of reviewing the MEMS adherence data in 9/16 of the intervention group. Found both the patient and provider found it 'helpful' in the moderate range (1 = not at all, 5= extremely) (patient 3.9 ± 0.93; provider 3.4 ± 1.2). Ratings from both indicated they were not at all 'uncomfortable' reviewing the adherence feedback - mean = 1. |
| Rosen et al., 2007 | Not assessed | |
| Ruppar, 2010 | N/a | Participants reported a sense of benefit from the intervention - particularly from the feedback components. Reported the primary benefit was from adherence and BP feedback. The participants looked forward to receiving information about their adherence level and BP control. Many intervention participants would compare their adherence and BP levels with those from their prior visit to gauge their progress and the need for further attention to their medication-taking behaviour. |
| Russell et al., 2011 | N/a | Participants felt the intervention took "very little" or the "right amount" of time. |
| Sabin et al., 2010 | Not assessed - though the monitor use did require a moderate degree of training. Basics required a full day of training and practice with local staff. After the first month of implementation, both staff and patients were comfortable with the technical details of monitor use. | Sustaining the intervention was not deemed burdensome. |
| Smith et al., 2003 | Not assessed | |
| Sulaiman et al. 2018 | Not assessed | |
| Sutton et al., 2014 | Not assessed | |
| Tashkin et al., 1991 | Not assessed | |
| van Onzenoort et al., 2012 | Not assessed | |
| Vasbinder et al. 2017 | Not assessed | |
| Velligan et al., 2013 | 5% in PharmCAT vs 38% MM complained about the treatment (P=0.012) - primary complaint was beeping of the device. | In 21 PharmCAT and 16 MM patients, satisfaction scores (1 not at all to 7 extremely satisfied) were higher in the PharmCAT (6.80 +/- 0.54) than MM (6.10 +/- 1.61; P=0.11). |
| Wilson et al., 2010 | Not assessed | |
| Wu et al., 2006 | Most common reasons for not using DMAS was leaving at home, two reported this was usually when they went out. Anecdotal feedback that the device was too large and too loud, resulting in unwelcome questions and possible exposure of HIV status. | When it beeped, most listened to the message; 57% never skipped in the past 4 days at week 4, 75% in week 20; 3 skipped the message 4 or more times in the past 4 days at week 4, and 1 at week 20. |
| Yeh et al., 2017 | Not assessed | |
